# Montelukast for bronchiolitis obliterans syndrome after lung transplantation: A randomized controlled trial

**DOI:** 10.1371/journal.pone.0193564

**Published:** 2018-04-06

**Authors:** David Ruttens, Stijn E. Verleden, Heleen Demeyer, Dirk E. Van Raemdonck, Jonas Yserbyt, Lieven J. Dupont, Bart M. Vanaudenaerde, Robin Vos, Geert M. Verleden

**Affiliations:** 1 KULeuven and UZ Leuven, Dept. of Clinical and Experimental Medicine, Division of Respiratory Diseases, Lung Transplant Unit, Leuven, Belgium; 2 KU Leuven, Department of Rehabilitation Sciences, Leuven, Belgium; UCLA, UNITED STATES

## Abstract

Bronchiolitis obliterans syndrome (BOS) remains the major problem which precludes long-term survival after lung transplantation. Previously, an open label pilot study from our group demonstrated a possible beneficial effect of montelukast in progressive BOS patients with low airway neutrophilia (<15%), and already on azithromycin treatment, in whom the further decline in pulmonary function was attenuated. This was, however, a non-randomized and non-placebo controlled trial. The study design is a single center, prospective, interventional, randomized, double blind, placebo-controlled trial, with a two arm parallel group design and an allocation ratio of 1:1. Randomization to additional montelukast (10 mg/day, n = 15) or placebo (n = 15) was performed from 2010 to 2014 at the University Hospitals Leuven (Leuven, Belgium) in all consecutive patients with late-onset (>2years posttransplant) BOS ≥1. Primary end-point was freedom from graft loss 1 year after randomization; secondary end-points were acute rejection, lymphocytic bronchiolitis, respiratory infection rate; and change in FEV_1_, airway and systemic inflammation during the study period. Graft loss at 1 y and 2y was similar in both groups (respectively p = 0. 981 and p = 0.230). Montelukast had no effect on lung function decline in the overall cohort. However, in a post-hoc subanalysis of BOS stage 1 patients, montelukast attenuated further decline of FEV_1_ during the study period, both in absolute (L) (p = 0.008) and % predicted value (p = 0.0180). A linear mixed model confirmed this association. Acute rejection, lymphocytic bronchiolitis, respiratory infections, systemic and airway inflammation were comparable between groups over the study period. This randomized controlled trial showed no additional survival benefit with montelukast compared to placebo, although the study was underpowered. The administration of montelukast was associated with an attenuation of the rate of FEV_1_ decline, however, only in recipients with late-onset BOS stage 1.

## Introduction

Lung transplantation (LTx) is the ultimate treatment option for selected patients suffering from end-stage pulmonary disorders [1] Mortality rates after LTx remain relatively high, mainly due to the high prevalence of chronic lung allograft dysfunction (CLAD) [2]. The term CLAD is nowadays used to describe all causes of irreversible decline in pulmonary function, including restrictive allograft dysfunction (rCLAD (restrictive CLAD)/RAS) [3] and the classical bronchiolitis obliterans syndrome (BOS) [4]. The incidence of BOS is about 10% per year, with a prevalence of 30% and 50% at 3 and 5 years, respectively. BOS is the single most important cause of late mortality accounting for 25–35% of all-cause mortality [5], increased morbidity, loss of quality of life and increased use of health care resources [6].

Treatment and prevention of BOS remains difficult. Initially, BOS was only treated by changing or increasing the immunosuppressive regimen, resulting, at best, in a temporary stabilization of the decline in forced expiratory volume in 1 second (FEV_1_) [7,8]. Recently it was demonstrated in a randomized controlled trial (RCT) that prophylactic azithromycin improved pulmonary function and reduced BOS prevalence at 2y after LTx [9]. Moreover, Corris et al. showed that azithromycin also improves lung function in a significant proportion of patients with established BOS, compared to placebo [10]. Other treatment options, such as total lymphoid irradiation (TLI) [11–13] and extracorporeal photopheresis (ECP) [14] proved to be of some benefit in the treatment of patients with BOS. Some positive effects of alemtuzumab (anti-CD52) in CLAD have also been reported [15].

A possible beneficial effect of montelukast was previously demonstrated in an open-label pilot study in BOS patients (mainly BOS stage I) with low bronchoalveolar lavage (BAL) neutrophilia (< 15%), in whom the further decline in pulmonary function despite azithromycin treatment was arrested [16]. Montelukast is a leukotriene receptor antagonist (LTRA), which possesses anti-inflammatory effects, especially on eosinophilic inflammation [17] and is thought to impact on airway remodeling. Moreover, animals models could also demonstrate beneficial effects of montelukast in the treatment of pulmonary fibrosis [18]. Finally, an observational study showed promising effects of montelukast in improving pulmonary function in 3 out of 5 patients with graft versus host disease (GVHD) after bone marrow transplantation, a condition very similar to BOS after lung transplantation [19].

In the present randomized controlled trial, we aimed to confirm the survival benefit of montelukast compared to placebo in BOS patients, with progressive FEV_1_ decline, despite azithromycin treatment (azithromycin non-responders).

## Material and methods

### Trial design

This was an investigator-driven, single center, prospective, interventional, randomized, double-blind, placebo-controlled trial in a tertiary hospital setting (University Hospitals Leuven). Eligible patients were included by their treating transplant physician (L.J.D., G.M.V., R.V, J.Y.). After written informed consent, patients were randomly assigned to receive montelukast (10 mg/day) or placebo (inclusion from November 2010 until July 2014). LTx recipients received add-on of study-drug (over-encapsulated montelukast or placebo) until the end of the study-period, additional to ‘standard of care’ treatment of BOS including azithromycin, 250 mg three times a week. None of the patients were treated with ECP at BOS diagnosis. Study groups were generated through permuted-block randomization using a 1:1 (in chronological order 1 = MLK and 0 = placebo: 1,0,0,1,0,1,1,0,1,1,0,1,0,1,0,1,1,0,1,1,0,0,0,0,1,1,0,0,0,1) ratio by the University’s Hospital Experimental Pharmacy (http://www.randomization.com). All participants, nurses, and treating physicians were blinded to group assignment during the study treatment and later follow-up. Evaluation of outcomes was performed after completion of the 1-year post initiation follow-up of the last included study patient in a blinded manner by investigators of the Leuven University Lab of Respiratory Diseases (D.R., S.V.). The data and safety monitoring board (Clinical Trial Center) and local Ethical committee (UZ Leuven, Leuven, Belgium: ML-6739 and S54604) approved the study (clinicaltrials.gov identifier: NCT01211509). After initiation, the treatment was continued (also after 2 years) and after unblinding, patients who had received placebo, were switched to open label montelukast. During the study period no changes in the trail design were made. The switch to open-label treatment was considered as an end-point for the initial intention-to-treat analysis. In case of further decline in FEV_1_, TLI was initiated in 1 patient despite open label montelukast.

### Study population

All adult (≥18 years) lung transplant recipients diagnosed with chronic rejection (BOS grade ≥ 1 and non-responsive to azithromycin (no increase in FEV_1_ to at least 80% compared to the 2 best post operative values) after at least 3 months of therapy, excluding azithromycin–responsive allograft dysfunction (ARAD) (increase in FEV_1_ to at least 80% compared to the 2 best post operative values) with signed informed consent and the ability to take oral medication were considered for enrolment in the study. Exclusion criteria were: retransplantation (lung), previous transplantation (solid organ), rapid decliner (decline in FEV_1_ of >150 ml/month during 3 months before inclusion), early onset of BOS (first 2 years after LTx) and multi-organ transplantation (lung + other solid organ). Diagnosis of chronic rejection (BOS grade ≥1) already excludes other potential confounding factors of chronic FEV_1_ decline [4].

After inclusion, patients were routinely (at least every 3 months) seen in our out-patient clinic. Pulmonary function testing was performed and blood was analyzed for routine biochemical markers. Before inclusion, a bronchoscopy with bronchoalveolar lavage (BAL) (2x50ml of saline) with cell count/differentiation, bacterial, fungal and viral evaluation, and histological evaluation of transbronchial biopsies was performed. After inclusion, bronchoscopy was only performed in case of suspected infection or acute rejection.

### Administration of investigational medicinal product

Montelukast (10 mg hard gelatine capsule formulation) was obtained from TEVA (Wilrijk, BE). For placebo, lactose monohydricum Ph. Eur. was purchased from Fagron (Rotterdam, the Netherlands) and compounded into hard gelatine capsules by the Leuven University's Hospital Experimental Pharmacy. Study medication was provided in numbered containers to the patients during routine follow-up visits at the outpatient clinic or during hospital admissions by a study nurse who also verified compliance and possible adverse events at each contact. Patients were instructed to continue treatment for 1 year. If a patients’ lung function further declined (100ml/month for more than 3 months after initiation), the study drug was stopped (without deblinding of the investigational drug) and the patient was initiated on open-label treatment with montelukast at 10 mg daily.

### End-points

Primary end-point was graft loss (mortality or retranspantation) at 1 and 2 year after inclusion. Secondary end points included acute rejection (AR) and lymphocytic bronchiolitis (LB) which were defined according to the ISHLT guidelines [20], during the study period within one year after inclusion. AR/LB was analyzed as a binary variable by contrasting at least one AR/LB event during follow-up versus no event. Another secondary endpoint was the presence of pulmonary infection (clinical symptoms of infection such as fever, dyspnea with an elevated C reactive protein (CRP) with/without chest X-ray changes and positive cultures (blood/sputum) with need for antibiotics), as well as change in FEV_1_ during the study period. Lastly, BAL cellular differential (airway inflammation) and CRP (systemic inflammation) were analyzed. Phenotypes of CLAD (rCLAD ((restrictive CLAD)/RAS) and the classical BOS) were diagnosed and differentiated according to histology, allograft function and imaging [21]. During the study period no changes in the end-points were made.

### Statistical analysis

Group means were compared using unpaired t-tests or Mann–Whitney tests for normally or non-normally distributed variables, respectively (Graphpad Prism 4.0 software, San Diego, CA, USA). Chi-squared test was used to compare proportions. Kaplan–Meier estimates with log-rank tests were used for time-to-event analyses. The rate of change in lung function was estimated using a linear mixed model analysis assuming random intercept and including intervention group as class variable (PROC mixed, SAS 9.4 statistical software). Fev_1_ (absolute and %pred measured at randomization and 3, 6 and 12 months later) was included as dependent variable. The main model included time [linear change (ml/month)], group and time*group interaction effect. Additionally, to investigate the possible effect modification by BOS stage (< X vs ≥X), we stratified the linear mixed model by BOS stage and tested the three way interaction time*group*BOS. No interim analysis were made before unblinding the study.

## Results

### Patients' characteristics

**Fig 1:**
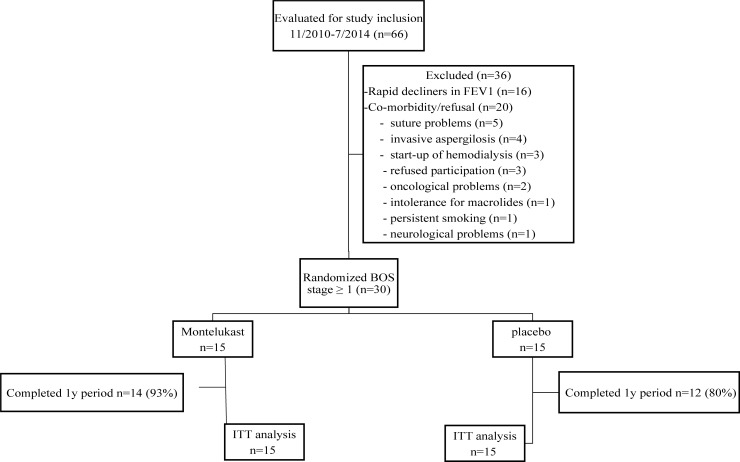
Flow chart of the randomized controlled trial of montelukast versus placebo in LTx patients with BOS. IIT = intention to treat analysis.

During the study period, a total of 66 patients were considered for inclusion. Thirty-six patients were not included (Fig 1). Most (n = 16) of these patients had a rapid decline in FEV_1_ (>150 ml/month). The other patients (n = 20) were not included due to co-morbidities or refusal (suture problems, meaning strictures or stenosis at the suture that influence allograft function, n = 5), invasive aspergillosis (n = 4), start-up of hemodialysis (n = 3), refused participation (n = 3), oncological problems (n = 2) and the single cases of intolerance for macrolides (n = 1), persistent smoking (n = 1), neurological problems (n = 1).

Thirty included patients were randomly assigned (placebo n = 15; montelukast n = 15) (Fig 1). Baseline characteristics were similar in both groups, as was immunosuppressive management (Table 1).

**Table 1:** Patient characteristics of the montelukast and placebo group. Values are presented as n-value (percentage) or mean (standard error of mean). AZA = azathioprine, BMI = body mass index, BOS = bronchiolitis obliterans syndrome, CF = cystic fibrosis, CLAD = chronic lung allograft dysfunction, CRP = C-reactive protein,CsA = cyclosporine A, FK = tacrolimus, HLTx = heart-lung transplantation, ILD = interstitial lung disease, MMF = mycophenolate mofetil, n = n-value, PHT = pulmonary arterial hypertension, POD = post-operative day, SLTx = single lung transplantation, SSLTx = double lung transplantation.

	Montelukast	Placebo
Subjects (n)	15	15
Recipient age (years)	61 (±1.7)	60 (±1.7)
Male/female	6/9	6/9
CLAD diagnosis (years after LTx)	5.0 (±0.6)	4.8 (±0.9)
Inclusion after diagnosis of BOS (days)	99 (±220)	88 (±138)
Underlying disease (n,%)		
- COPD/Emphysema	11 (73)	11 (73)
- ILD	2 (13)	2 (13)
- PHT	1 (7)	2 (13)
- CF	1 (7)	0 (0)
Type of transplantation (n,%)		
- SSLTx	11 (73)	11 (73)
- HLTx	0 (0)	1 (7)
- SLTx	4 (27)	3 (20)
Immunosuppressive treatment (n)		
- FK/CsA	14/1	11/4
- AZA/MMF/none	7/4/4	7/5/3
Stage of BOS at inclusion (n,%)		
- BOS 1	8 (53)	11(73)
- BOS 2	6 (40)	3 (20)
- BOS 3	1 (7)	1 (7)
BMI (kg/m^2^)	25.5 (±1.4)	25.2 (±1.2)
Duration of treatment (days)	333 (±53)	401 (±38)
Time on Azithromycin (days) before study inclusion	466 (±99)	518 (±175)
Reason start Azithromycin (n,%)		
- ARAD	5 (33)	7 (47)
- Declining lung function	8 (54)	5 (33)
- Post-operative/infectious	2 (13)	3 (20)
Drop-out (n,%)	1 (7)	3 (20)
- Gastro-intestinal intolerance	0 (0)	1 (7)
- Malignancy	1 (7)	1 (7)
- Withdrawal of informed consent	0 (0)	1 (7)
CRP (mg/L)	5.8 (±2.3)	2.7 (±0.9)
BAL cell differentiation at BOS diagnosis		
- Neutrophilia, %	21.3 (±7.9)	22.4 (±8.0)
- Total neutrophilia, x10^6^/ml	0.08 (±0.05)	0.10 (±0.07)
- lymphocytosis, %	6.1 (±1.9)	8.3 (±1.9)
- Total Lymphocytosis, x10^6^/ml	0.007 (±0.004)	0.010 (±0.003)
- eosinophilia, %	0.74 (±0.36)	0.4 (±0.15)
- Total eosinophilia, x10^6^/ml	0.0003 (±0.0001)	0.002 (±0.001)
- Total leucocytes, x10^6^/ml	0.016 (±0.06)	0.17 (±0.08)

A total of 12/15 (80%) of the patients in the placebo group and 14/15 (93%) of the patients in the montelukast group completed the 1-year study drug treatment period (p = 0.598). Reasons for discontinuation are given in Table 1. Discontinuation was initiated either by the patients (i.e. patients tolerated the study drug but withdrew from the study (n = 1), gastro-intestinal intolerance (nausea) (n = 1)) or by the investigators (study medication was stopped due to supportive care in patients with diagnosis of a malignancy (n = 2)). Three (20%) patients of the placebo group and one (7%) of the montelukast group were initiated on open-label montelukast treatment after further decline in FEV_1_ (p = 0.598). Additionally, Table 2 summarizes the characteristics of BOS stage 1 patients in the montelukast and the placebo group. No differences could be identified between both groups.

**Table 2:** Patient characteristics of the montelukast and placebo group (BOS stage I only). Values are presented as n-value (percentage) or mean (standard error of mean). AZA = azathioprine, BMI = body mass index, BOS = bronchiolitis obliterans syndrome, CF = cystic fibrosis, CLAD = chronic lung allograft dysfunction, CRP = C-reactive protein, CsA = cyclosporine A, FK = tacrolimus, HLTx = heart-lung transplantation, ILD = interstitial lung disease, MMF = mycophenolate mofetil, n = n-value, PHT = pulmonary arterial hypertension, POD = post-operative day, SLTx = single lung transplantation, SSLTx = double sided lung transplantation.

	Montelukast	Placebo
Subjects (n)	8	11
Recipient age (years)	59 (±2.9)	59 (±2.1)
Male/female	4/4	5/6
CLAD diagnosis (years after LTx)	5.0 (±1.1)	4.2 (±0.8)
Underlying disease (n,%)		
- COPD/Emphysema	6 (74)	7 (64)
- ILD	1 (13)	2 (18)
- PHT	1 (13)	2 (18)
- CF	0 (0)	0 (0)
Type of transplantation (n,%)		
- SSLTx	7 (87)	7 (64)
- HLTx	0 (0)	1 (9)
- SLTx	1 (13)	3 (27)
Immunosuppressive treatment (n)		
- FK/CsA	7/1	9/2
- AZA/MMF/none	3/2/3	6/3/2
BMI (kg/m^2^)	26.5 (±1.8)	26.7 (±1.0)
Time on Azithromycin (days) before study inclusion	615 (±316)	408 (±112)
CRP (mg/L)	3.1 (±1.0)	9.3 (±4.2)
BAL cell differentiation at BOS diagnosis		
- neutrophilia, %	17.0 (±7.1)	16.0 (±8.8)
- Total neutrophilia, x10^6^/ml	0.021 (±0.01)	0.096 (±0.09)
- lymphocytosis, %	4.3 (±1.2)	7.3 (±1.9)
- Total Lymphocytosis, x10^6^/ml	0.004 (±0.001)	0.009 (±0.004)
- eosinophilia, %	0.80 (±0.67)	0.47 (±0.19)
- Total eosinophilia, x10^6^/ml	0.009 (±0.001)	0.002 (±0.001)
- Total leucocytes, x10^6^/ml	0.13 (±0.26)	0.17 (±0.10)

### Primary end-points

#### Graft loss

**Fig 2:**
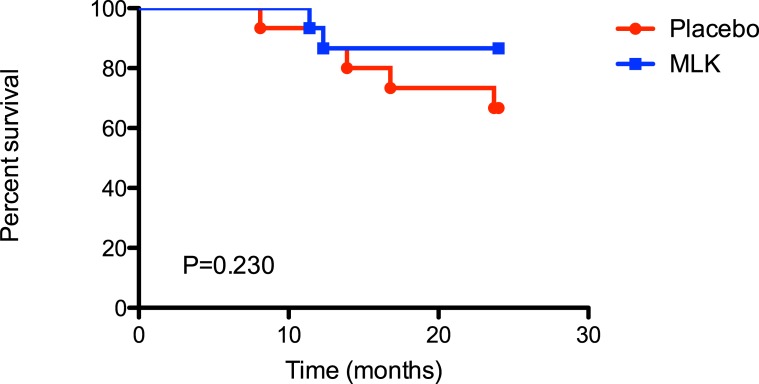
Kaplan-Meier survival curve of the placebo and the montelukast arm at 2 years.

Graft loss in both groups was similar at 1 year (p = 0.981), and much lower than expected. One patient (6.5%) died in both groups. Both patients died because of respiratory failure due to end-stage BOS, without concurrent infection. None of the included patients were re-transplanted during the time of the study. Also, 2-years mortality rate did not statistically differ (p = 0.230) (montelukast (2/15, 13%) and placebo (5/15, 33%) (Fig 2).

### Secondary end-points

#### Lung function evolution

**Fig 3:**
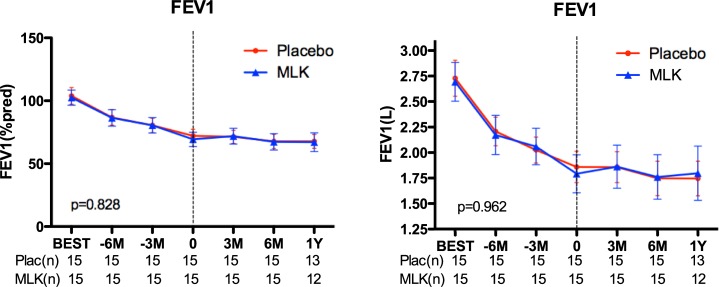
FEV_1_ evolution (% predicted, absolute value) comparing montelukast to placebo. MLK = montelukast. Dotted line is the time-point of inclusion.

No difference in lung function was observed between both groups neither at respectively at six and three months before inclusion in the study nor or at the time of the initiation of the study medication. Lung function evolution after inclusion was also similar in both groups (p = 0.962 and p = 0.828 respectively for absolute value (L) and % predicted value) (Fig 3). Linear mixed model showed respectively p = 0.315 and p = 0.4626.

Lung function evolution of all individual cases is shown in S1 Fig. In Table 3, patients are categorized according to lung function evolution: stabilization (110%-90% compared to the time of BOS diagnosis), improvement (>110%) and deterioration (<90%). Similarly, no difference was observed between both groups (p = 0.096). In both groups, 1 patient with an initial BOS phenotype converted to the RAS phenotype during the study period (evolution from an obstructive spirometry with only air-trapping on CTscan to a restrictive lung function with persistent infiltrates due to fibrosis on CTscan).

**Table 3:** Long function evolution of the montelukast and placebo group. Patients were subdivided in stable (110%-90% FEV1 compared to the FEV1 at the moment of CLAD diagnosis), increase (>110%) and decrease (<90%), no difference was observed (p = 0.16). No result = no result could be obtained in 2 patients in the placebo group and 3 patients in the montelukast groups due to mortality, concurrent infection, missed appointment. MLK = montelukast.

PLACEBO, n		3 Months	6 Months	12 months
	Improvement (>110%)	3	1	1
	Stabilization (90%-110%)	8	9	6
	Deterioration (<90%)	4	5	6
	No result	0	0	2
MLK, n				
	Improvement (>110%)	4	5	1
	Stabilisation (90%-110%)	10	4	7
	Deterioration (<90%)	1	6	4
	No result	0	0	3

**Fig 4:**
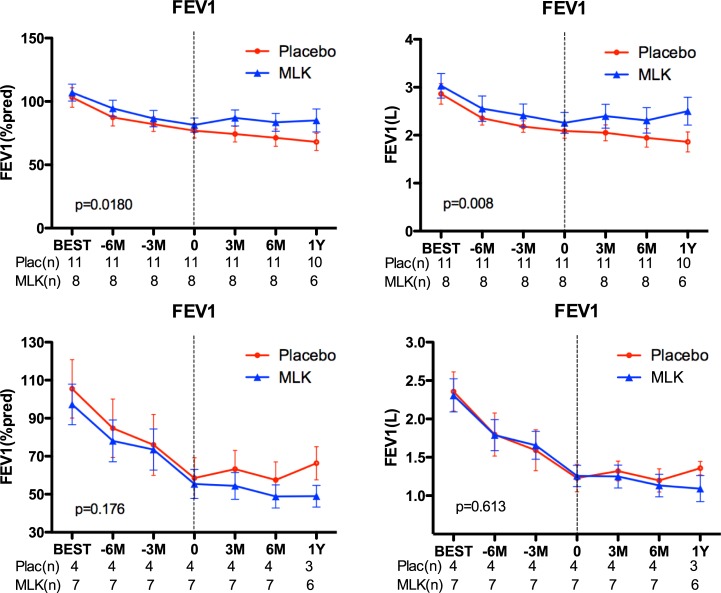
FEV_1_ evolution (% predicted, absolute value) comparing montelukast to placebo in BOS stage 1 patients (upper part) and BOS stage 2 and 3 (lower part). MLK = montelukast. Dotted line is the time-point of inclusion.

In a post-hoc analysis, we only analyzed the patients in BOS stage 1. In both groups, patients were predominantly in BOS stage 1 at study drug initiation, 11/15 (73%) and 8/15 (53%) patients in the placebo and the montelukast group respectively (p = 0.479). In this early BOS stage, a significant effect of montelukast on FEV_1_ evolution could be demonstrated, both in absolute value (L) (p = 0.008) and % predicted value (p = 0.018) (Fig 4). The Linear mixed model showed respectively p = 0.0682 and p = 0.0339. At 6 months, 4 out of 8 (50%) BOS stage 1 patients in the montelukast study arm improved their FEV_1_, while none in the placebo group (p = 0.0181). In later BOS stages (2–3), only one patient in each group showed an improvement (S2 Fig). In BOS 1 patients, we observed a stabilization of FEV_1_ in the montelukast group (+9 (±14) ml/months), while in placebo group the FEV_1_ further declined (-24(±14) ml/month) (p = 0.198) (S3 Fig).

### Acute rejection and lymphocytic bronchiolitis

In the montelukast group a mean of 0.60 (±0.91) and in the placebo group a mean of 1.13 (±1.30) bronchoscopies were performed during the first year after inclusion (p = 0.228). No acute rejection episodes were observed in either group during the time of the study period. In the placebo group, 3 patients developed one or more episodes of lymphocytic bronchiolitis, during the study period, compared to none of the patients in the montelukast group (p = 0.224). In the placebo group, 4 patients developed ≥1 respiratory infections, compared to 2 patients in the montelukast group (p = 0.651).

### Airway and systemic inflammation

BAL cellularity and plasma CRP, assessed at inclusion in the study, were comparable in both groups (p = 0.937 and p = 0.229, respectively). BAL cellular differentiation is also shown in Table 1. During the follow-up at 3, 6, 9 and 12 months, CRP was comparable between both groups (p = 0.937).

### Adverse effects

No serious adverse effects were observed, only one patient in the placebo group stopped the treatment after 5 days, due to nausea.

## Discussion

BOS remains the most important cause of late morbidity and mortality after LTx. Montelukast previously suggested promising results in a pilot study in BOS patients, demonstrating attenuation of the decline in FEV_1_ compared with a retrospectively matched control group [16]. However, the question remains whether this is a natural evolution of BOS or a real drug effect? The current randomized controlled trial trial with montelukast could not reveal a decrease in graft loss compared to placebo after the diagnosis of BOS, at least within 2 years of treatment initiation. As a consequence, our primary endpoints showed no survival benefit, although the study was underpowered with respect to the primary endpoints. Also secondary end points showed non-superiority, although in a post–hoc analysis (linear mixed modelling), additional montelukast (10 mg/day) in BOS stage 1 patients seemed to have beneficial effects on the FEV_1_ decline during the study period of 1 year, compared to placebo patients in whom the FEV_1_ further declined.

Despite this possible novel finding in BOS patients not responding to azithromycin, our results confirm that not all LTx patients with BOS may equally benefit from a treatment with montelukast, but rather that this drug may be started as soon as BOS is diagnosed and certainly when patients are still in stage 1. It remains questionable whether MLK might have an effect in early BOS (within 2 y after transplantation) and in rapid decliners, as these were excluded from our study. Groups were small, underpowered for the primary end-point, and these results should be interpreted with caution and need confirmation in a larger number of patients.

Freedom from graft loss in both groups, was comparable, which may be due to initiation of open-label montelukast treatment in patients with progressive BOS during the study period, possibly resulting in better long-term outcome in those patients. As such, at the end of study period, 3/15 patients of the placebo arm (20%) had been initiated on open-label montelukast treatment. Also, the finding that graft loss was not significantly different between both groups is actually in line with previous reports demonstrating that patients with BOS tend to live longer when treated with azithromycin [22], and the study period of one year was probably too short to see a survival benefit. Overall, our 1 y mortality after onset of BOS was very low, as only 2/30 patients (6.5%) died. Nevertheless, the 2 y mortality in the placebo group was 33%, versus 20% in the MLK group. Although not significantly different, there seems to be a beneficial signal compared to the Finlen-Copeland study [23] where 2 y mortality in late onset BOS patients was >30% (only 18 patients at risk), despite the fact that also single lung transplantation with a worse post BOS survival, was included in our study (22 patients at risk).

The exact mechanism of action of montelukast remains to be investigated. However, in animal models, leukotriene receptor antagonists (LTRA) are able to inhibit pulmonary fibrosis [17]. Whether cysteinyl leukotrienes are present in the airways in patients with BOS remains unknown, but cysteinyl leukotrienes are accepted to be involved in airway remodeling, such as basement membrane thickening in asthmatics [23] and in fibroblast proliferation [24], which may offer a possible explanation for the effect of montelukast in BOS 1 patients, in whom there is probably ongoing remodeling early after disease onset.

The efficiency of montelukast was also observed in the treatment of BOS after allogenic hematopoetic stem cell transplantation, a disease comparable with BOS after LTx. As already mentioned in the introduction, the study of Or et al [19] shows beneficial effects of montelukast in improving pulmonary function in 3 out of 5 patients with graft versus host disease (GVHD) after allogenic hematopoetic stem cell transplantation. Another single-agent, open-label study evaluated montelukast in 25 patients with established BOS after allogenic hematopoetic stem cell transplantation and showed stabilization with less than 15% decline in FEV1 for the entire cohort over 6 months of treatment [25]. Also in combination therapy (with fluticasone and azithromycin (FAM) or combination with budesonide/formoterol and n-acetylcysteine), montelukast shows improvement of lung function in patients with BOS after allogenic hematopoetic stem cell transplantation [26,27].

Another important observation, is that no serious adverse effects were observed in all patients treated with montelukast which was generally well tolerated. This confirmed the observations made by Or et al [19]. Moreover, it might by far be the cheapest current treatment option available for patients with BOS stage 1.

There are, however, many drawbacks of this study. This is a single center study, which explains the long duration of inclusion with a low number of patients. Yet, power was too low to reach statistically significant differences, especially concerning the primary end point of graft loss. At the time of writing the protocol (2009–2010), not many data on survival of BOS patients was available. This may have lead to false assumptions, making our study not robust enough because this study was underpowered to reach the primary end-points. Indeed, based upon available literature at the time, we estimated a graft loss of 30% at 1 year, which was used in our power analysis to calculate the number of patients needed in each group. Since mortality was much lower in both groups, (only 6.5%), the power of this study is much too low to show a difference in graft loss. Also the study of Corris et al. [10], which is the only available RCT in the treatment of BOS, was underpowered to reach the primary end-points. As a consequence, these 2 studies could be exemplary in the need for multi-center studies in order to have sufficient power (sample size) to reach the defined end-points. The prevalence of BOS during the recruitment period was also below what was previously estimated which, in retrospect, is probably due to the beneficial effects of azithromycin. Consequently, our recruitment rate was on average one per 1.5 month rather than the estimated one per month. Therefore, our recruitment period was 42 months rather than the anticipated 36 months. Also, at the time of study initiation, no knowledge yet existed concerning the heterogeneity of CLAD and the relatively fast evolution in adapted treatment of the different phenotypes (TLI, ECP, montelukast, perifinidone,…), making time bias possible. Nevertheless, at time of inclusion, no single patient had the RAS phenotype and only 2 BOS patients evolved to RAS during follow-up. The role of montelukast in early RAS patients remains to be further determined. We have also taken into account that the study was performed in patients with a late diagnosis of BOS (mean time of diagnosis around 5y after LTx), and it is known that late-onset BOS patients have a better prognosis than rapid decliners and early-onset BOS, were no medical treatment seems to improve the outcome [22,28].

It is unknown from this study why not all BOS 1 patients show a benefit from montelukast. Although the characteristics of responding and non-responding patients seem to be no different, the number of patients is too low to enable further evaluation. This clearly deserves attention in a large multicenter RCT. In the natural history of BOS, FEV1 very often stabilizes without any intervention, as also seen in the placebo arm of the current study [29]. As a consequence, the results of the post-hoc analysis should be interpreted with caution.

In conclusion, montelukast has no effect on lung allograft survival within the constraints of the limited power of this study. Compared to placebo, administration of montelukast, showed some attenuation of the rate of FEV_1_ decline in recipients with late-onset BOS stage 1, not responding to azithromycin. This conclusion definitely needs further investigation in a multicenter study, because of the low number of included patients.

## Supporting information

S1 CONSORT Checklist (DOC)

S1 Fig Individual lung function (FEV1) evolution (absolute value, % predicted), upper part placebo and lower part montelukast group. (DOCX)

S2 Fig **Lung function (FEV1) evolution (absolute value, % predicted) comparing montelukast to placebo of BOS stage 1 patients (upper part) and BOS stage 2 and 3 (lower part)**. MLK = montelukast. Dotted line is the time-point of inclusion.
(DOCX)

S3 Fig **Lung function (FEV1) evolution (% predicted) comparing montelukast to placebo of BOS stage 1 patients (upper part) and BOS stage 2 and 3 (lower part)**. MLK = montelukast. Dotted line is the time-point of inclusion. FEV1 in the montelukast group (+9 (±14) ml/months), while in placebo group the FEV1 further declined (-24(±14)ml/month) (p = 0.20)
(DOCX)

S1 Protocol (PDF)

S1 File Data. (XLSX)

## Abbreviations

AR: Acute rejection
ARAD: Azithromycin–responsive allograft dysfunction
BAL: Bronchoalveolar lavage
BOS: Bronchiolitis obliterans syndrome
CLAD: Chronic lung allograft dysfunction
CRP: Reactive protein
ECP: Extracorporeal photopheresis
FEV_1_: Forced expiratory volume in 1 second
GVHD: Graft versus host disease
ISHLT: International society of heart and lung transplantation
LB: Lymphocytic bronchiolitis
LTRA: Leukotriene receptor antagonist
LTx: Lung transplantation
RAS: Restrictive allograft dysfunction
RCT: Randomized controlled trial
TLI: Total lymphoid irradiation
