# Supplementary material for: Montelukast for bronchiolitis obliterans syndrome after lung transplantation: A randomized controlled trial
Source: PLoS One. 2018 Apr 6;13(4):e0193564. doi: 10.1371/journal.pone.0193564 (PMC5889063; doi:10.1371/journal.pone.0193564)
Supplement: S1 Fig — (DOCX) [file pone.0193564.s002.docx]

Figure S1

**Figure S1:** **Individual lung function (FEV_1_) evolution (absolute value, % predicted), upper part placebo and lower part montelukast group**.
