# Supplementary material for: Montelukast for bronchiolitis obliterans syndrome after lung transplantation: A randomized controlled trial
Source: PLoS One. 2018 Apr 6;13(4):e0193564. doi: 10.1371/journal.pone.0193564 (PMC5889063; doi:10.1371/journal.pone.0193564)
Supplement: S3 Fig — Lung function (FEV1) evolution (% predicted) comparing montelukast to placebo of BOS stage 1 patients (upper part) and BOS stage 2 and 3 (lower part). MLK = montelukast. Dotted line is the time-point of inclusion. FEV1 in the montelukast group (+9 (±14) ml/months), while in placebo group the FEV1 further declined (-24(±14)ml/month) (p = 0.20) (DOCX) [file pone.0193564.s004.docx]

Figure S3

**Figure S3.** **Lung function (FEV1) evolution (absolute value) comparing montelukast to placebo of BOS stage 1 patients (upper part) and BOS stage 2 and 3 (lower part).** MLK= montelukast. Dotted line is the time-point of inclusion.
