# Supplementary material for: Montelukast for bronchiolitis obliterans syndrome after lung transplantation: A randomized controlled trial
Source: PLoS One. 2018 Apr 6;13(4):e0193564. doi: 10.1371/journal.pone.0193564 (PMC5889063; doi:10.1371/journal.pone.0193564)
Supplement: S1 Protocol — (PDF) [file pone.0193564.s005.pdf]

IWT-TBM application 2010

Leuven Lung Transplant Unit

Prof Geert M Verleden

A novel treatment strategy to tackle chronic rejection after lung transplantation.

PART I: PROJECT IDENTIFICATION

Title of the project:

A novel treatment strategy to tackle chronic rejection after lung transplantation.

**Start date of the project:**	January 2011
**Project duration requested:**	four years
**Requested budget:**	848.403 euro
**Manpower:**	3 x 48 = 144 mm
**%budget for the hospital:**	20% of the budget

Main Applicant

Prof Geert M verleden Geert.Verleden@uzleuven.be

Head of the Lung Transplant Unit at UZ Gasthuisberg, Leuven, Belgium

national ID 590-7984628-02

Name project leaders

Research Laboratory of Pneumology

Dr Bart M Vanaudenaerde Bart.Vanaudenaerde@med.kuleuven.be

Stijn Verleden Stijn.verleden@med.kuleuven.be

University Pharmacy

Dr Apo Hutsebaut Lisbeth Lisbeth.hutsebaut@uzleuven.be

Dr Apo Kim De Gieter Kim.degieter@uzleuven.be

Dr Saskia Vandenput Saskia.vandenput@uzleuven.be

Annemie Peys Annemie.Peys@uzleuven.be

Research Laboratory of Endocrinology KULeuven

Prof. Dr. R. Bouillon Roger.bouillon@uzleuven.be

Prof. Dr. C. Mathieu Chantal.Mathieu@uzleuven.be

This research group is linked with both the university (KULeuven) and the hospital (UZLeuven)

PROJECT SUMMARY

Lung transplantation is an accepted therapeutic option for patients with an end-stage lung disease. In Belgium a yearly average of 90 lung transplantations are performed (60% in Flanders). The long-term survival remains far below other solid organ transplantation with a median survival of only 5 years (1). This is attributed to chronic rejection or Bronchiolitis Obliterans Syndrome (BOS), which has an incidence of 10% per years and a prevalence of 50% after 5 years. Chronic rejection is the most important cause of late mortality after lung transplantation and leads to enormous morbidity and use of health care resources (2;3). Few therapeutic options are available. Recently, we described a dichotomy within chronic rejection with a Neutrophilic Reversible Allograft Dysfunction (NRAD) phenotype and a Fibroproliferative BOS (fBOS) phenotype (4;5). Together with other groups, we demonstrated that NRAD accounts for 40% of chronic rejection and can be treated with the neo-macrolide azithromycin On the other hand, the remaining 60% of the patients not responding to azithromycin, "the fBOS phenotype", have no neutrophilic inflammation. The current idea is that fibroproliferation alone drives this rapid deteriorating fBOS (6).

With this IWT-TBM project we aim to **decrease chronic rejection (with 25%) and related mortality (with 15%)** by tackling both phenotypes of chronic rejection: firstly, **preventing neutrophilic inflammation with high dose Vitamin D**, and secondly **treating fibroproliferation (fBOS) with montelukast.** Our proof of concept indicates the potential of this therapeutic strategy. Therefore, we will conduct **two prospective, double-blind, randomized, placebo-controlled clinical trials** to have the ultimate proof allowing to move forward to implementation in standard care for lung transplant patients.

In the first study, 100 patients will be randomised to Vitamin D or placebo after 1 month of transplantation and followed up for 2 to 3 years for the development of chronic rejection. In the second study, 30 patients with chronic rejection (fBOS) will be randomized to montelukast or placebo and followed for 1 to 2 years to evaluate survival and chronic rejection progression.

The project output will improved prevention and interventional therapy for chronic rejection after lung transplantation, which will: 1) decrease the incidence of chronic rejection, the progression of chronic rejection; 2) improve the long-term survival; 3) improve the quality of life and 4) decreased the direct and indirect use of health care resources.

PART II. PROJECT DESCRIPTION

A. POSITIONING TOWARD THE TBM PROGRAMME

Few therapeutic options are available to treat chronic rejection and only azithromycin has proven to be really effective in 40% of affected patients (7). Moreover existing treatments are very expensive and not available in every centre. As a consequence, more intensive research is definitely needed to make further progress in the battle against chronic rejection after lung transplantation. Based on our current knowledge on chronic rejection, with its specific phenotypes and mechanisms, we want to target the most important mechanistic features of the two major phenotypes. **It is our intention to prevent and to treat chronic rejection with adequate and tailored treatment strategies.** Adding basic research will allow us to elucidate new mechanistical insights and perhaps explore new diagnostical tools again further tailoring the treatment strategy.

In this study we will focus on montelukast and vitamin D, as we **recently observed a beneficial effect in a pilot study** (a delay in the disease progression of chronic rejection) of montelukast in 11 patients with chronic rejection non-responsive to azithromycin. Regarding vitamin therapy we observed that 60% of our lung tranplant patients have a 25-OH vitamin D deficiency. **This project includes 2 parallel double blind placebo controlled randomized trials** which may deliver absolute proof of its potential, allowing to progress to implementation in daily routine patient care and treatment.

In Belgium, around 800 lung transplantations have been performed since the first lung transplantation in 1969 by Prof F Derom. Nowadays about 90 lung transplantations are performed per year, of which **50 in UZleuven with a centre total of over 550**. So the UZLeuven lung transplant centre, one of the top 10 most active lung transplant centres, represents a well positioned group and even the only one in Flanders/Belgium able to conduct this study and also able to later on implement this new strategy if succesfull. Despite the fact that lung transplantation and hence, the development of chronic rejection, is rather infrequent compared with for instance COPD or asthma, the yearly costs are high. In Belgium (Flanders=UZLeuven) the yearly cost for lung transplantation is about 29.6

million (17.7 million) euro of which 5.0 million (3.0 million) euro accounts for chronic rejection. These costs are enormous for an orphan disease but can be attributed to the frequent hospitalisations, extensive immunosuppressive treatment regimens and use of different diagnostic procedures (8;9). The impact of our previous research has already changed the concepts, the diagnostic procedures and the treatment strategies of chronic rejection not only in Belgium but also in the rest of the world. Our results with azithromycin have also changed concepts beyond transplantation in the respiratory field. Currently a large clinical trail with azithromycin (AZISAST) in severe asthma is recruiting patients and basic research towards the potential of azithromycin to treat idiopatic pulmonary fibrosis is being conducted.

Despite all our efforts and encuraging results, it remains frustrating to obtain enough funding since lung transplantation is **an "orphan" disease**. Also the new study strategy of **using "old, off label" medication already present for many years** in other fields of (pulmonary) medicine (with generic drugs present on the market) prohibits interest from pharmaceutical companies. Our intention is only to improve patient care and to lower the health care costs. As a consequence, industrial interest is low or even non existing.

Because of all these arguments, we are confident that our project does fullfill all the criteria of the TBM programme.

B1. RATIONALE OF THE PROJECT

With this IWT-TBM project we will use a totally new treatment strategy to tackle the prevalence of chronic rejection and to treat it by interfering with both mechanisms involved namely neutrophilic inflammation and fibroproliferation.

With a median survival of 5 years, long-term survival for lung transplant patients remains far below other solid organ transplantation (10). This can mainly be attributed to the development of chronic rejection or Bronchiolitis Obliterans Syndrome (BOS). The incidence of chronic rejection is about 10% per year with a prevalence of 30% and 50% at 3 and 5 years post transplantation (Table I). Chronic rejection is the single most important cause of death accounting for 25-35% of the late mortality (Table I). Moreover, it leads to increased morbidity, loss of quality of life and increased use of health care resources (11).

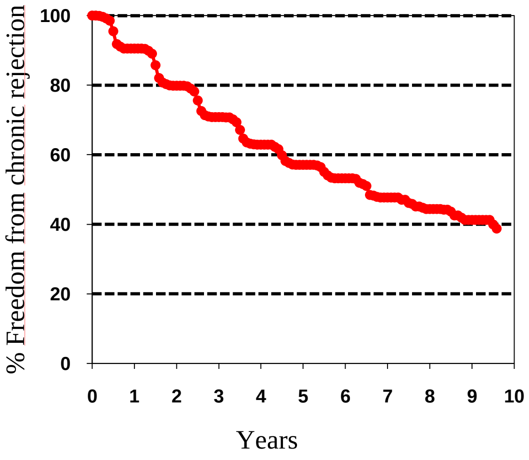

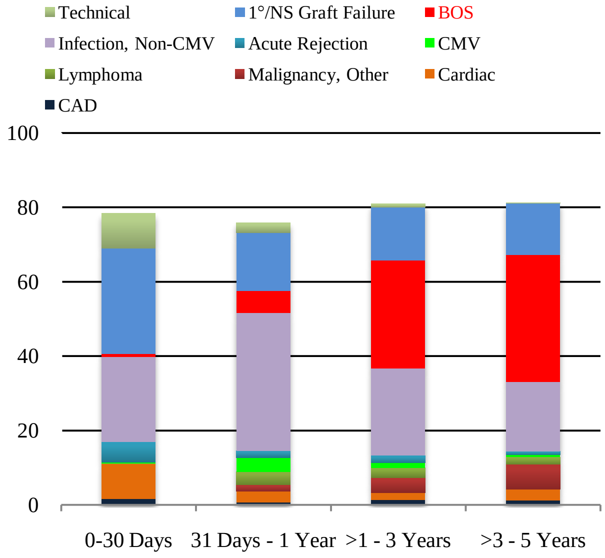

Table I: The freedom of chronic rejection (BOS)(left) and the cause of mortality (right) according to the international society for heart and lung transplantation (12).

Few therapeutic options are available to treat chronic rejection and only azithromycin has proven to be really effective in some 40% of affected patients (13). Moreover existing treatments (such as photopheresis and total lymploid irradiation) are very expensive and not available in every centre. As a consequence, more intensive research is definitely needed to make further progress in the battle against chronic rejection after lung transplantation.

The basis of this new therapeutic strategy is our recent findings of a dichotomy within chronic rejection. Up to recently chronic rejection was regarded as one homogeneous entity including two major elements: first, an abnormal inflammatory response of the airways and second, a fibroproliferative obliteration of these airways. Chronic rejection was clinically recognized by a persistent cough, sputum production and dyspnea and functionally assessed by a progressive loss of forced expiratory volume in one second (FEV_1)(14). Opposed to the current believe, we have demonstrated that these elements (neutrophilic inflammation and fibroproliferation) are the hallmark of two totally different phenotypes of BOS, a Neutrophilic Reversible Airways/Allograft Dysfunction (NRAD) and a Fibroproliferative Bronchiolitis Obliterans Syndrome (fBOS)(Table II)(15). The best discriminating factor is the response to the neo-macrolide azithromycin, which only improves the NRAD phenotype.

	**Neutrophilic reversible allograft dysfunction (NRAD)**	**Fibroproliferative (fBOS)**
**Inflammation (BAL)**	neutrophilic airway inflammation	no airway inflammation
**Clinically**	coarse crackles and increased sputum production	no coarse crackles, minimal sputum
**Time of onset**	onset early after transplantation	onset late after transplantation
**Progression**	slow progression (several years)	rapid progression (half a year)
**Histology**	initially inflammatory, ends up in pure fibrosis	pure fibrosis
**Radiology**	bronchiectasis, airway wall thickening, mucus plugging	air trapping and consolidation
**Azithromycin**	reversible (effective)	irreversible (ineffective)

Table II: The dichotomy within chronic rejection or Bronchiolitis Obliterans Syndrome after lung transplantation (16).

To further progress in the battle against chronic rejection, we need to address as well neutrophilic airway inflammation as well as fibropoliferation. The inflammation needs to be picked up and treated before the diagnosis of chronic rejection is made, at least when NRAD is concerned. With respect to the fBOS phenotype, we do not know yet what triggers the onset, but we do know that it may progress very fast with a steep decline in FEV_1. As a consequence, the best we can do is trying to slow-down the progression.

Besides the known risk factors for the development of chronic rejection (HLA-mismatch, acute rejection episodes, receptor age, CMV and other viral infections), we and other

groups recently described new risk factors all acting via induction of a neutrophilic airway inflammation. These new risk factors are Pseudomonal colonisation, lymphocytic bronchitis/bronchiolitis, gastro-oesophageal reflux, air pollution, smoking and ischemia-reperfusion injury (17-27). We also identified new markers of chronic rejection (the pH of exhaled breath condensate, exhaled carbon monoxide, airway neutrophilia and systemic C-reactive protein)(28-30), which are all linked with innate airway inflammation with a prominent role for neutrophils (=NRAD phenotype). Patients who experience more neutrophilic inflammation after transplantation, become more prone to develop the NRAD phenotype of chronic rejection,. Therefore, new therapies that potentially prevent or treat this neutrophilic airway inflammation are mandatory, not only to reduce the severity but also the number of these events, which eventually may lower the prevalence of chronic rejection.

Current treatment strategies of chronic rejection have little effect and could at best arrest the deterioration of the FEV_1 and certainly do not discriminate for this dichotomy (31;32). Only azithromycin has recently been demonstrated to have a beneficial effect on the FEV_1 evolution (33). Most of the current therapeutic agents focus on the adaptive immunity (lymphocytes) and on the fibroproliferation respons. Yet, most of these agents are toxic and associated with a lot of side-effect. Rapamycin, for instance, is a new anti-fibrotic agent which was not able to arrest the progression of chronic rejection, however, it was associated with an increase in infectious episodes (34). We rather believe that drugs with little or no side-effects such as azithromycin that specifically treat the innate inflammation and perhaps also affect fibroproliferation, are new targets we should focus on. Such drugs should not necessarily be newly designed agents, it is possible that renewed interest in some agents can be more useful (with azithromycin as the proof of the pudding). Therefore, we will focus on the potential role of Vitamin D and montelukast in prevention and treatment of chronic rejection.

The rational for the present study is based on this concept of the dichotomy, using a treatment and prevention option that better targets the 2 major mechanistic elements: neutrophilic inflammation and fibroproliferation. On top of this we want to use therapeutic agents which are less toxic, have few side-effects and are less expensive.

B2. STATE OF THE ART/ NOVELTIES

Vitamin D is generally accepted to be important in bone metabolism and calcium housekeeping. The choice to investigate Vitamin D as a potential chronic rejection-preventive drug is based on two important findings: firstly, it is know that mild vitamin D deficiency (revealed by low serum 25-OHD levels) is present in more than 50% of patients with advanced pulmonary disease and correlates with FEV_1 decline (COPD patients) and increased susceptibility for microbial infections (35). Secondly, vitamin D exerts pleotropic effects fully covering newly described risk factors for BOS. Indeed, vitamin D down-regulates neutrophilic inflammation, oxidative stress, autoimmune activation, T_H17 differentiation and bacterial colonisation (36;37).

As proof of concept for using vitamin D in lung transplantation, we investigated vitamin D deficiency in COPD (GOLD4) patients in UZ Leuven which are candidates to be listed for transplantation and account for almost 50% of the lung transplantation indications in our centre: 97% of these severe COPD patients had a deficiency in serum levels of vitamin D. Furthermore, we also investigated 55 lung transplant patients during routine yearly follow-up and we observed a deficiency in 60% of these patients, demonstrating its potential use in the prevention of chronic rejection (figure I). A large study with vitamin D treatment in renal transplantation aiming to prevent early complications such as acute rejection, infection and CRP levels is currently ongoing in Austria (clinicaltrials.gov NCT00752401), which further illustrates the potential role of vitamin D within transplantation.

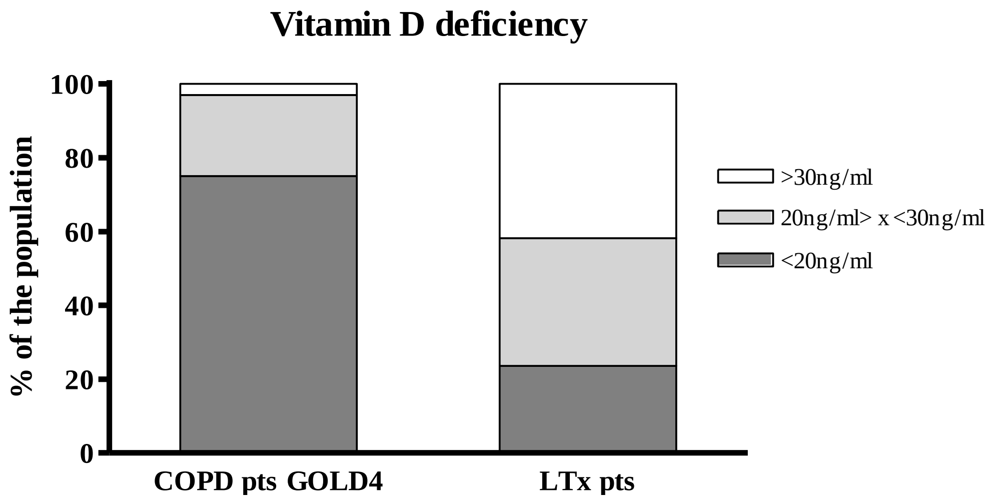

Figure I: Vitamin deficiency (<30 ng/ml) determined in the COPD (GOLD4) and lung transplant patients at UZLeuven.

The second aim of the study is to slow-down the progression of chronic rejection, especially the fBOS phenotype. As the present understanding of this phenotype is rather poor, there is a need for intensive research on this most lethal and frequent phenotype of chronic rejection, with a median survival of about 50% at 2 years after diagnosis (Figure II).

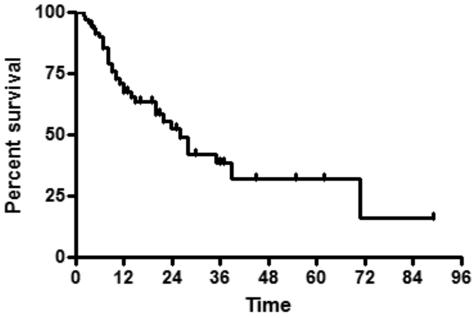

Figure II: Survival of fBOS patients after diagnosis (Leuven experience, n=72)

Leukotrienes may emerge as a mechanistic target, since these mediators act as proliferating agents and seem to be elevated in BAL fluid of lung transplant patients (own results, not yet published). Leukotrienes (LTs) are lipid mediators derived from the arachidonic acid metabolism and can be divided in LTA4, LTB4 and the cysteinyl-LTs (LTC4, LTD4 and LTE4). LTA4 and LTB4 are involved in inflammation but cysteinyl-LTs (cysLTs) are more related to fibrosis mechanisms. CysLTs directly affect migration, proliferation, and extracellular matrix protein synthesis by fibroblasts (38). To target these elements we are interested in the use of montelukast, which is a cysteinyl-leukotriene1 receptor antagonist. Montelukast is already widely used in the treatment of asthma where it reverses airway remodelling, including subepithelial collagen deposition (39). It has some anti–inflammatory effects, especially on eosinophilic inflammation (40), but is also known as an antiproliferative agent (41;42). Moreover, in a recent pilot study montelukast improved pulmonary function in 60% of the patients with graft versus host disease after bone marrow transplantation, which is pathologically characterized as obliterative bronchiolitis, the hallmark of fBOS (43). As a consequence, a large NCI driven study with montelukast for OB associated with graft versus host disease after bone marrow or stem cell transplantation is ongoing in the States (clinicaltrials.gov NCT00656058).

To maximise the effect of montelukast, it is of crucial importance to start the therapy as early as possible after chronic rejection has been diagnosed. Therefore, randomization will occur as soon as fBOS is diagnosed (BOS grade 1 and a BAL neutrophilia <15%). We hypothesize that administration of montelukast may arrest the fibroproliferation and stabilize chronic rejection (and hence arrest the decline in FEV_1). As a consequence, we aim to reduce the BOS-related mortality. Mechanistical studies will accompany this clinical trial to give further insight into the underlying mechanisms allowing us to move forward in the treatment of this most devastating phenotype of chronic rejection.

As proof of concept, we investigated 22 lung transplant patients diagnosed with the fBOS phenotype of chronic rejection of which 11 patients received additional montelukast therapy and compared them to the 11 patients on standard therapy (figure III). In the montelukast group an arrest in the FEV_1 decline from 112±26 mL/month before BOS diagnosis to 13±13 mL/month (p=0.001) 6 months after diagnosis was observed. In the control group, we found no change in the rate of FEV_1 decline: 103±20 mL/month before BOS diagnosis versus 114±17 mL/month (p=0.74) after diagnosis was observed. The decline in FEV_1 during 6 months of montelukast treatment was different compared to the FEV_1 decline of the control group in the same period (p=0.001), whereas the 6 month decline before chronic rejection was not different between the control and montelukast group (p=0.74). This study is submitted for publication.

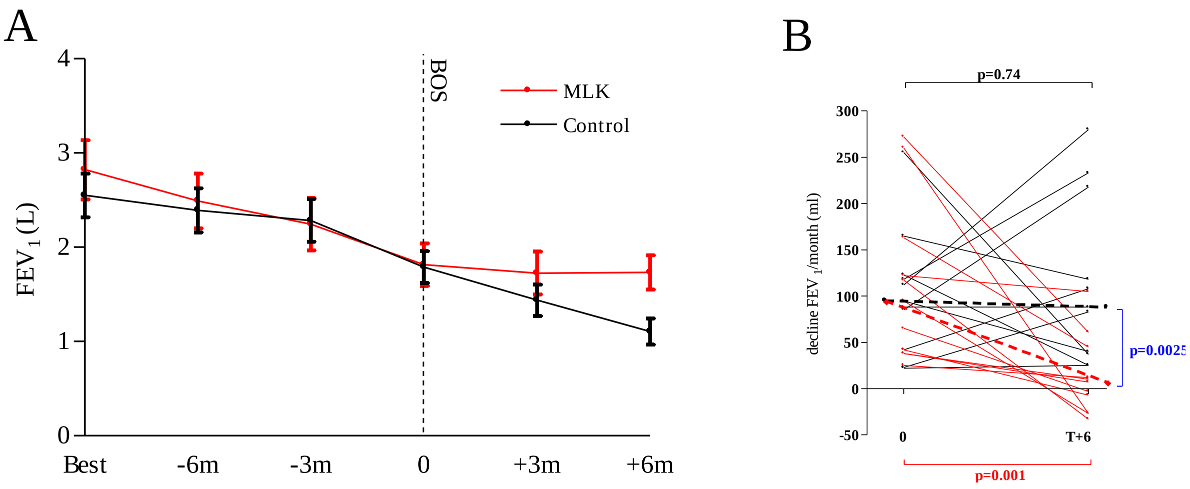

Figure III: Within a cohort of 22 lung transplant patients diagnosed with chronic rejection (fBOS) 11 were treated with montelukast (MLK). After the start of the study drug, the decline in FEV_1 was arrested (p=0.001) in the MLK group while the control group showed no difference (p=0.74). The decline after diagnosis of fBOS was significant lower in the montelukast group (p=0.0025).

This project does not concern patents at all. Also this project has no connection with any other ongoing IWT project.

In summary: The vitamin D study starting just after lung transplantation is based on the presence of vitamin D deficiency within severe COPD patients (candidates to be listed for transplantation and representing 60% of the transplant population) and within a cohort of our own lung transplant patients. We hypothesize that vitamin D can downregulate important triggers like reflux, autoimmune reaction, dendritic cells, IL17 producing T cells, finally leading to a decreased airway neutrophilia and consequently a reduction of the prevalence of chronic rejection (especially the NRAD phenotype). Second, we will treat lung transplant patients diagnosed with chronic rejection (BOS grade 1 and 2, but not 3) with montelukast to arrest the fibroproliferation and deterioration of the disorder (specifically the fBOS phenotype). It is our goal to find an optimal tailored treatment strategy for both phenotypes.

B.3 INNOVATION/AIMS

The primary goal of this project is to decrease the prevalence of chronic rejection and hence to increase the long-term survival after lung transplantation.

First aim:	**Vitamin D substitution to prevent chronic rejection**<br>**Reduce chronic rejection with 25%**
Second aim:	**Montelukast as rescue treatment for chronic rejection (fBOS)**<br>**Reduce the chronic rejection related mortality with 15%**

Within these treatment strategies we will investigate clinical outcome measures, BAL biopsies and blood to understand the underlying mechanisms and to find risk factors and early markers allowing even earlier and better diagnosis.

To study the therapeutic effect of Vitamin D and montelukast in the prevention and treatment of chronic rejection after lung transplantation we will conduct two parallel prospective placebo-controlled double blind randomised studies in lung transplanted patients. Furthermore, we want to explore the mechanisms of action of both Vitamin D and montelukast and the mechanism of chronic rejection. The combination of the clinical trial and the mechanistical research will allow us to explore whether Vitamin D and/or montelukast tackle one specific phenotype of chronic rejection allowing us to introduce a more tailored treatment strategy.

The duration of both studies is calculated to be 4 years with a reanalysis at 5 years. Considering that the start of the study will be in October/November 2010 this project is conforming the duration range of the TBM period.

A major advantage of this project is the unique and well controlled setting of lung transplantation. A routine follow-up with BAL, lung biopsies and blood starting at the time of transplantation allows us to study the phases before, at the time, and after the onset of chronic rejection and this even within the lung itself. These findings can open easily perspective for other types of transplantation and chronic lung disease where invasive sampling (other organs or severe disease stage) of early disease (like pulmonary fibrosis, chronic pulmonary disease...) is difficult to study.
The proof of concept study with the vitamin D deficiency in lung transplant population and the response to montelukast treatment for the fBOS phenotype indicates that this novel therapy strategy will likely be efficient for lung transplant patients.

Unique to our research centre, the study and the treatment strategy is that if this study would be successful, it will be instantaneously without any delay implemented in the standard patient care and treatment in our centre. One could consider government refunding as an issue but one needs to consider that these treatments are available as generics and moreover these therapies are mostly less expensive compared to other commonly used and experimental immunosuppressive therapies.

De doelstelling van dit project is het verlagen van chronische afstoting en de daarmee gepaard gaande mortaliteit na long transplantatie.

Deel 1:	**Vitamin D supplementen om chronische afstoting te voorkomen**<br>**De prevalentie van chronische afstoting 25% doen afnemen**
Deel 2:	**Montelukast als behandeling van chronische afstoting (fBOS)**<br>**De chronische afstoting gerelateerd mortaliteit met 15% doen afnemen**

Binnen deze studie willen we klinische parameters alsook BAL, biopsies en bloed onderzoeken om inzicht te krijgen in onderliggende mechanismen en risico factoren en vroegtijdige merkers van chronische afstoting om zo een nog betere behandeling en vroegtijdige diagnose te kunnen bekomen.

C. UTILISATION OF THE RESULTS

C.1.IMPACT OF THE STUDY

Our aim is to validate the therapy for preventing chronic rejection with vitamin D and treating chronic rejection (the fBOS phenotype) with montelukast as new gold standard of care in lung transplantation.

Our findings will have an immediate socio-economic impact.

As mentioned before, despite being an orphan disease, lung transplantation and chronic rejection cause a lot of morbidity/mortality and use of health care resources (see above). Yearly costs to transplant and to treat BOS are high and very well demonstrate the socio-economic impact on the health care resources and the justification of this project on lung transplantation.

The direct impact **of vitamin D** (25% reduction chronic rejection) would be estimated to be a **reduction of 1.25 (0.75) million euro in Belgium (Flanders=UZ Leuven) per year** related to the reduction in prevalence of chronic rejection. The impact of **montelukast** (reduction of 15% in mortality) **exceeds any calculation in terms of financial impact**. These are only the direct impact and the indirect elements are not even considered like the quality of life which will increase due to the lower frequency of chronic rejection. This can lead to a higher percentage of the population being implemented in normal live as opposed to the situation now where most patients do not return to work after their transplantation, or stop working especially when they develop chronic rejection.

One could consider government refunding as an issue but one needs to consider that these treatments are available as generics and moreover **these therapies are less expensive compared to other commonly used and experimental immunosuppressive therapies**.

C.2. THE UTILISATION OF THIS PROJECT:

All lung transplant patients could benefit from the implementation of vitamin D to the standard care to prevent chronic rejection, and even if they do develop chronic rejection the patients can benefit from the effect of montelukast to stabilize the progression of chronic rejection. In this way we aim to improve the overall survival and quality of life of our lung transplantation cohort.

Our project will have an immediate medical impact for the individual patient.

If this study would be successful, the results would be **instantaneously implemented, without any delay or hurdles to be taken, in the standard therapy in our centre.** This is the biggest advantage of our lung transplant research centre where research findings can be without any delay translated to patient care and treatment, and that is why this project should be well considered for funding.

In Belgium approximately 800 lung transplantations have been performed so far, of which more than 550 in UZLeuven. With an average of about 50 lung transplantation per year, our centre is one of the top 10, most active transplant centres worldwide. As UZLeuven performs >95% of the lung transplantation in Flanders and about 60% of the transplantations in Belgium the largest proportion is easily reached. But these findings will go further as we are used to communicate our findings nationally and internationally by presenting our results at yearly national and international meetings in the transplantation and respiratory field: the annual Belgian Transplant Society (BTS), the annual Belgian Respiratory Society meeting (BVP), the annual meeting of the International Society of Heart and Lung Transplantation (ISHLT), the American Thoracic Society (ATS) and the European Respiratory Society (ERS) meeting. It is, however, our goal to publish in international peer reviewed papers and to reach the international lung transplantation society to transfer our findings to all transplant centres in a non-profit way. Over the last 20 years, findings have resulted in numerous publications in top end journals of the respiratory field (AM J Respir Crit Care Med) and the transplantation field (Am J Transplant). Our research has changed the concepts and treatment in lung transplantation. The idea that 40% of patients with chronic rejection can be adequately treated with azithromycin and the existence of a dichotomy, is gaining more and more international appreciation. The prevalence of chronic rejection 5 years after transplantation has decreased from 50% to 30% in UZ Leuven, proving that our findings are also beneficial for our transplant patients.

Prof GM Verleden is also currently chairman of the lung transplant group of the European Respiratory Society, past chairman of the Pulmonary Council and current board member of the International Society of Heart and Lung Transplantation, co-chair of an international task force on lung transplantation/BOS of the ATS/ERS/ISHLT, reflecting the impact of the Leuven Lung Transplantation Group worldwide.

It would be our goal to reach the same level with this study as we were able to reach with our findings on azithromycin which is now becoming standard therapy not only at UZLeuven and in Belgium but also world-wide.

Besides these direct utilisation potential also indirect potential needs to be considered as chronic rejection after lung transplantation serves as a model for other chronic lung disorder. Our results with azithromycin have also been translated to other disorders like COPD, idiopatic pulmonary fibrosis, severe asthma (the AZISAST study) and bone marrow transplantation making the potential impact of our research on health care enormous.

C.3. INTELLECTUAL PROPERTY RIGHTS OF THE PROJECT

This project is **not associated with own patent or intellectual rights**.

There is **freedom to operate for the utilization of the project results**.

Vitamin D is a generic compound and patent coverage (US 5,565,473 and the derived family) for Singulair expires on August 3, 2012. Vitamin D and or Singulair have not been claimed in a medicinal claim for transplant rejection.

D. RESEARCH APPROACH AND WORK PROGRAMME

This project will start in Oktober/November 2010 and is a combination of 2 clinical studies: one to prevent chronic rejection by administration of high dose of Vitamin D and the other to treat chronic rejection (the fBOS phenotype) by administration of montelukast. A schematic presentation is shown in figure IV.

During and after the study-period, usual immunosuppressive treatment will be given to all patients: rATG during 3 days, cyclosporin or tacrolimus, azathioprine or mycophenolate mofetil and methylprednisolone (according to standardized, routine immunosuppressive protocol). Similarly, anti-infective prophylaxis for all patients will consist of co-trimoxazole (2 days per week, lifelong), inhaled amphotericin B for the first 1 to 3 months, aciclovir or ganciclovir for 3 months (according to standardized, routine prophylactic protocol)(44).

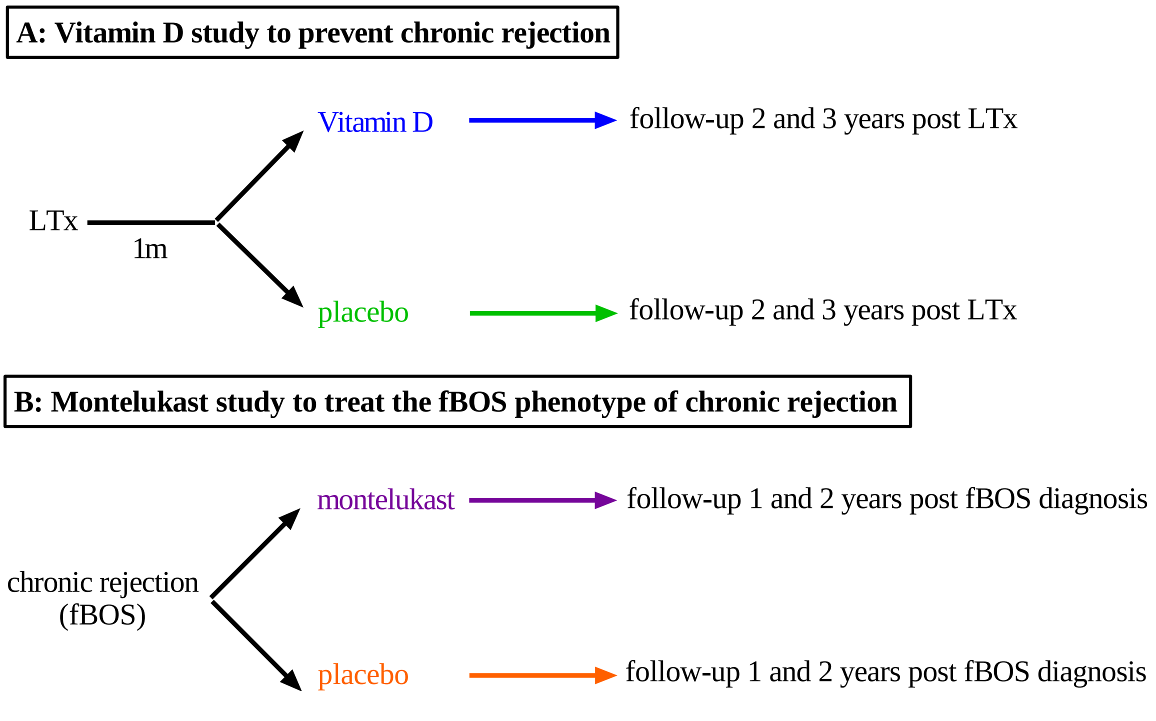

Figure IV: Schematic presentation of the study design of the clinical trials.

D1: A PREVENTION STUDY WITH HIGH DOSE VITAMIN D SUPPLEMENTS

For the prevention study with Vitamin D, after informed consent, patients will be randomised one month after transplantation to the vitamin D (D-Cure, 100.000 units orally/month) or placebo arm of the study. A monthly dose of vitamin D is ideal as the half-life time of vitamin D (Cholecalciferol) is about 2 months and observation from a COPD trial, using the same dosing, rarely demonstrated toxic levels within the blood (45). The advantage of a monthly dose is that it can be given to the patient at the routine monthly visit to the outpatient clinic to prevent non-compliance. This is a single centre prospective, interventional, randomized, double-blind, placebo-controlled trial in a clinical setting (tertiary University Hospital), which is investigator-driven and without a pharmaceutical sponsor. Lung transplant recipients will receive add-on of study-drug (placebo or vitamin D) to 'standard of care', with a study-drug regime of 100.000 U/month until the end of the study-period. The placebo: vitamin D inclusion ratio is 1:1. All stable adult (age at least 18 years at the time of transplantation) lung transplant recipients at discharge after transplantation, with signed informed consent and the ability to take oral medication are considered to be enrolled in the study. Exclusion criteria are: prolonged and/or complicated ICU-course after transplantation; early (<30 days post-transplant) post-operative mortality, major suture problems (airway stenosis or stent), retransplantation (lung), previous transplantation (solid organ) and multi-organ transplantation (lung+ other solid organ).

Primary endpoint:

prevalence of chronic rejection (BOS grade 1) at 2 and 3 y post-transplant

Secondary endpoints:

BAL: cells (differential cell count, fibrocytes)
proteins (IL-8, LTD4 and so on; see below),
microbiology

Peripheral blood: CRP and fibrocytes

CMV and non-CMV infection rates

Acute rejection and lymphocytic bronchiolitis rates

Reflux (clinical and biochemical approach)

To have a statistical power of 80% ($\alpha = 0.05$; $\beta = 0.20$) to detect 25% reduction in chronic rejection (BOS1) after 2 years we estimate that 72 patients are needed. Considering the exclusion criteria (30%) and the drop-out (10%), a total population of 100 patients need to be enrolled in the study. The start of recruitment is October/November 2010. Important to mention is that, from previous experience, almost 100% of our patients are willing to participate in a study with a centre average of about 50 lung transplantations per year, the inclusion will take about 2 years. With a 2 and 3-year follow-up, the end of this part of the study will be reached after 4 to 5 years.

D2: A STUDY TO TREAT BOS WITH MONTELUKAST IN COMBINATION WITH AZITHROMYCIN

After informed consent, patients will be randomised at the time of diagnosis of chronic rejection (fBOS phenotype) to the montelukast (10 mg/day) or the placebo arm of the study.

This is a single centre prospective, interventional, randomized, double-blind, placebo-controlled trial in a clinical setting (tertiary University Hospital), which is investigator-driven and without a pharmaceutical sponsor. Lung transplant recipients will receive add-on of study-drug (over-encapsulated placebo or montelukast) with a study-drug regime of 10 mg/day of montelukast until the end of the study-period to ‘standard of care’ including azithromycin. The placebo: montelukast inclusion ratio is 1:1.

All adult (>18 years) lung transplant recipients diagnosed with chronic rejection (BOS grade 1 and non-responsive to azithromycin) with signed informed consent and the ability to take oral medication are considered to be enrolled in the study. Exclusion criteria are: retransplantation (lung), previous transplantation (solid organ) and multi-organ transplantation (lung+ other solid organ). Diagnosis of chronic rejection (BOS grade 1) already excludes potential confounding factors.

Primary endpoint:

> The survival /retransplantation rate at 1 and 2 y post-diagnosis of chronic rejection (BOS1)

Secondary endpoints:

Obstructive and restrictive pulmonary function evolution

BAL: cells (neutrophils, lymfocytes, fibrocytes)

proteins (RAGE, HGF, IL8, MMP8/9, LTD4 and others see below),

microbiology

Peripheral blood: CRP and fibrocytes

Acute rejection and lymphocytic bronchiolitis rates

CMV and non-CMV infection rates

Gastroesophageal reflux (clinical and biochemical approach)

To have a statistical power of 80% ($\alpha = 0.05$; $\beta = 0.20$) to detect a 15% reduction in survival rates after 1 and 2 years we estimate that 25 patients are needed. Considering the exclusion criteria (10%), a total population of 30 patients needs to be enrolled in the study.

The start of recruitment is November 2010 and with a centre average of 10 diagnosis of chronic rejection (specifically fBOS) per year, the inclusion will take about 3 years. Again, important to mention is that, from previous experience, almost 100% of our patients are willing to participate in a study. With a 1 and 2 -year follow-up, the end of this part of the study will be reached after 4 to 5 years.

D3. SPECIFIC DETAILS AND BASIC RESEARCH MODALITIES

As soon as the project will start patients will be included and randomised post transplant to the Vitamin D or placebo group or at diagnosis of chronic rejection to the montelukast group or to the placebo group. The lung transplant team will supply the study-drug to the patients via the routine follow-up at the outpatient clinic and at hospital admissions. There will be no additional costs for the patients or the hospital, since all examinations (except one) that will be performed (blood samples, lung function, X-rays, CT scans, fibre-optic bronchoscopies with BAL and biopsy) are already being used as routine follow-up parameters. The extra examination is the evaluation of the vitamin D trough level in blood (Ca, phosphate, PTH, 25-OHD, 1,25 $(OH)_2D$) and urine (Ca/creatinin ration, erythrocytes) sample. This extra investigation is accounted for in the project budget.

BAL will be performed at day 2, 30, 90, 180, 360 and then every 6 months as part of our routine post-transplant follow-up, which started routinely for every patient since 2001. Transbronchial biopsies (TBB) will be taken at day 30 and 90 and afterwards whenever there is a clinical indication (infiltrate on chest X-ray, suspicion of acute or chronic rejection,…), again as per our routine follow up protocol. At the time of diagnosis of chronic rejection a bronchoscopy with BAL and biopsy is performed to validate the diagnosis but also a follow-up bronchoscopy is planned 3 months later (if there is no routine follow-up) to evaluate the progression (response to azithromycin) routinely.
Bronchoscopic procedures (BAL) are used for histology and cell differentials, but also for microbiological/virological assessments and assessment of protein levels. BAL differential cell counts will be performed (% macrophages, lymphocytes, neutrophils and eosinophils) and protein-analyses. BAL microbiology (Chlamydia, Mycoplasma, Pseudomonas,...) will be assessed in the University Hospital Lab.

BAL will be analysed for different markers within the lab of pneumology to study different pathophysiological pathways that we assume to be involved: innate inflammation, fibrosis, angiogenesis, airway remodelling, epithelial damage and oxidative stress including CRP, bile acids, MMP9, TIMP, RAGE, MPO, IL8, MPO, VEGF, HGF, PDGF, TGFβ, IL1β and cysLT. These parameters will be analysed by ELISA, CBA and searchlight. The potential of these markers has already been investigated in a retrospective study of chronic rejection. Blood, BAL and biopsies will also be investigated on the presence of key immunological cells involved in chronic rejection like dendritic cells, regulatory T cells, IL17 producing T cells (TH17, NKT17,γδT17) and fibrocytes. Several risk factors will be evaluated within the study such as there are acute rejection episodes, lymphocytic bronchitis, infections, Pseudomonas colonisation, GER, smoking and several comorbidities.

Spirometry will be performed in agreement with American Thoracic Society (ATS)-criteria, prior to bronchoscopy (Masterscreen, Jaeger, Hoechberg, Germany). Chronic rejection will be diagnosed and BOS graded according to the International Society for Heart and Lung Transplantation (ISHLT) working formulation.

This routine follow-up does not need to be organised as it is already standard practice in our unit since 2001 again making our research unique and well placed to perform this project.

D4. SCHEMATIC OVERVIEW OF ESTIMATED MAN-MONTHS:

Estimated Personnel allocation (manmonths)		YEAR 1	YEAR 2	YEAR 3	YEAR 4
**Work package 1**<br>patient selection and inclusion<br>information and informed consent<br>information and guidance	PhD student<br><br>study nurse	1 mm<br><br>2 mm	1 mm<br><br>2 mm	/<br><br>/	/<br><br>/
**Work package 2**<br>collection of routine clinical information<br>Including medication compliance and side-effects	study nurse	7	7	9	9
**Work package 3**<br>organisation of routine follow-up<br>Bronchoscopy<br>BAL (with analysis)<br>Biopsies<br>Blood<br>pulmonary function	PhD student<br>study nurse	1mm<br>3 mm	1 mm<br>3 mm	0<br>3 mm	0<br>3mm
**Work Package 4**<br>processing and analysis of BAL, biopsy and blood<br>cell count,and flow cytometry<br>Microbiology<br>Protein measurement	technician	10 mm	10 mm	10 mm	10 mm
**Work Package 5**<br>vitamin D level measurements<br><br>Blood collection and processing	technician	2 mm	2 mm	2 mm	2 mm
**Work Package 7**<br><br>production of medication and placebo<br>medication delivery	Pharmacist<br>routine	routine	routine	routine	routine
**Work package 8**<br>Organisation, analysis of data and writings manuscripts	PhD student	10 mm	10 mm	12 mm	12 mm

D5. PROJECT BUDGET

Personeelskosten										
Functie	1st jaar		2e jaar		3e jaar		4e jaar		totaal	
	# mm	Kost/mm	# mm	Kost/mm	# mm	Kost/ mm	# mm	Kost/ mm	# mm	kost
PhD student (bursaal)	12	38169	12	39695	12	41440	12	43240	48	160544
Study nurse	12	53729	12	55404	12	55404	12	58855	48	225101
Technical unit	12	42280	12	43655	12	44719	12	47303	48	177957
**Total**	**36**	**134179**	**36**	**138754**	**36**	**138754**	**36**	**149398**	**144**	**565602**

Overige kosten: Optie 1 max. 50% van de totale personeelskosten; geen verdere detaillering vereist.					
**Overige kosten berekend als een percentage van de personeelskosten.** includes study medication, vit D measurements, ELISA, CBA, searchlight , immunohistochemistry, flowcytometry, consumables and indirect costs					
**50 % van de personeelskosten**	**67090**	**69377**	**71636**	**74699**	**282801**

Totale kosten voor het consortium	
**Personeelskosten**	565602
**Overige kosten**	282801
**Grote onderaannemingen**	/
**Totale kosten**	**848403**
**Totaal aantal mensmaanden**	**144**

E. POSITIONING OF THE APPLICANT AND FEASIBILITY

To be able to perform this study, a well running and experienced clinical and research unit is essential. Applying for such a project as a single centre approach may be very ambitious. Yet the 20 years of clinical experience and 10 years of basic research experience in lung transplantation have made this centre a top end and leading centre in the world.

This is also not our first experience with a large single centre randomised placebo controlled study as we recently conducted and published a large randomised placebo-controlled study with azithromycin (83 patients) which changed the therapy of lung transplant patients over the world as azithromycin is now part of the standard care of treatment. Prof Verleden is currently part of a task force to revise the definitions of Bronchiolitis Obliterans Syndrome (BOS).

Of course this study can only be achieved with the support of a large group of clinical and laboratory collaborators. The Leuven Lung Transplant unit consists of a large group of individuals with diverse backgrounds and functions that are well communicating and making our group so successful.

The Leuven Lung Transplant Team

Head of the Lung Transplant Research Group
Prof Dr. Geert M. Verleden

Project leaders
Dr Bart Vanaudenaerde
Stijn Verleden

PhD or MD students

Dr Robin Vos
Anna Willems-Widyastuti
Stephanie Devleeschauwer
Stijn Willems

Supporting Professors of the Research Group

Prof Dr. Marc L. Decramer
Prof Dr. Lieven J. Dupont
Prof Dr. Marion Delcroix
Prof Dr. Dirk E. Van Raemdonck
Prof Dr. Christophe Dooms

Lung Transplant Coordinators

Joachim De Roey
Dirk Claes
Glen Van Helleputte
Bruno Deschamps

Social and Phychological unit

Dirk Delva
Valentine Lemaigre

Lung Transplant Patient Care Unit

Annemie Schoonis
Christine Rosseel
Veronique Schraevers
Christel Jans
Mieke Meelberghs

Bronchoscopy Patient Care Unit

Joseph Foulon	Frie Van de Weyer
Linda Verschueren	Philippe Vandenbergh

Hospital pharmacia

Dr Lisbeth Hutsebaut	Dr Saskia Vandenput
Dr Kim de gieter	Annemie Peys

Laboratory of Endocrinology

Prof Dr Chanthal Mathieu
Prof Dr Roger Bouillon

The Leuven Lung Transplant team also gathered substantial research funding over the last 5 years allowing some stability in the research projects. This has also led to multiple collaborations nationally and internationally.

Project funding

Project from the Research Foundation Flanders (FWO): G.0493.04
229.000 euro; 1/1/2004 up to 1/1/2008

Project from the Research Foundation Flanders (FWO): G.0518.06
100.000 euro; 1/1/2007 up to 1/1/2011

Project from the Research Foundation Flanders (FWO): G.0643.08.
364.000 euro; 1/1/2008 up to 1/1/2012

Senior research fellowship (Bart Vanaudenaerde) of the Research Foundation Flanders (FWO)
225.000 euro; 1/1/2008 up to1/1/2011

PhD research fellowship (Robin Vos) of the Research Foundation Flanders (FWO)
224.000 euro; 1/10/2007 up to 30/9/2011

Extension senior research fellowship (Bart vanaudenaerde) of the research foundation flanders (FWO)
225.000 euro 1/1/2011 up to 1/1/2014

OT grant funded by the KULeuven
300.000 euro; 1/10/2010 up to 1/10/2014

GMV is holder of the Glaxo-Smith-Kline (Belgium) chair in respiratory pharmacology

50.000 euro/year; 1990 to 2011 and further

Collaboration with other groups national and international

A. Boehler and W. Weder

Dept of Pulmonary Medicine, University Hospital Zurich, Switzerland

Concerning the mouse lung transplant model

J.C. Hogg

The J Hogg iCapture centre, University of British Columbia, Vancouver, British Columbia, Canada

Concerning microCT and microarray analysis of human lungs

J. Hostens

SkyScan, Kontich, Belgium

Concerning microCT analysis

H.S. Sharma

Dept of Pharmacology, Erasmus MC, Rotterdam, the Netherlands

Concerning cell culture and angiogenesis

E.K. Verbeken

KULeuven, dept of pathology, Leuven, Belgium

Concerning histology for both human and animal tissue

T.S. Nawrot, P.H. Hoet and B. Nemery

University Hasselt, Diepenbeek, Belgium

KULeuven Dept of Public Health, Leuven, Belgium

Concerning the link air pollution and lung transplantation

W. Janssen, G.N. Gayan-Ramirez and M.L. Decramer

KULeuven, dept of pneumology, Leuven, Belgium

Concerning lung function measurement in animals

D. Lambrechts

Vesalius Research Centrum, Dept Moleculaire en Cellulaire Geneeskunde, Leuven, Belgium

Concerning target gene polymorphism measurements

P. Proost

KULeuven, Dept. Microbiologie en Immunologie, Leuven, Belgium

Concerning chemokine involvement in chronic rejection

In the end only two elements which need to be considered are funding and n-values (enough patients for clinical studies?). The question about the number of patients can be answered by the yearly numbers of lung transplantations and diagnosis of chronic rejection (as demonstrated in figure IV).

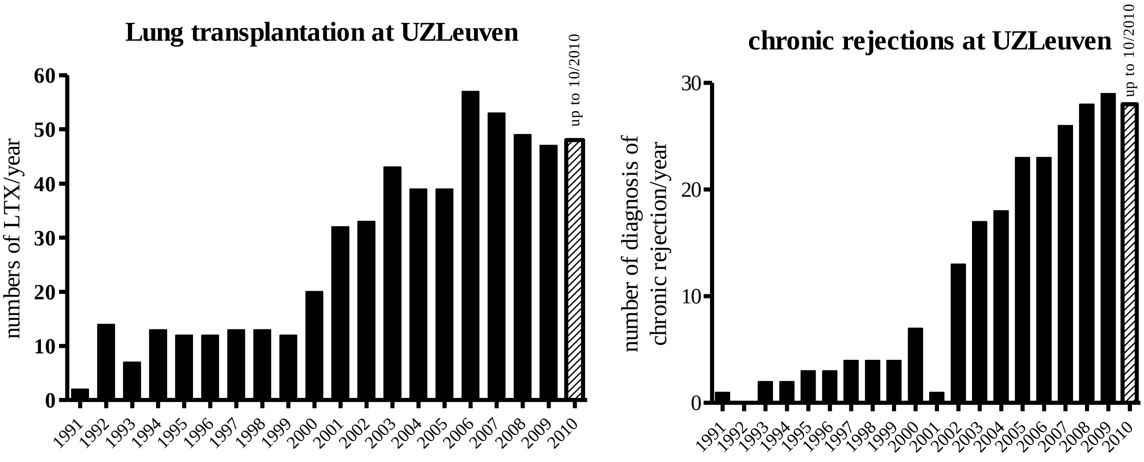

Figure IV: The yearly numbers of patients transplanted and the yearly diagnosis of chronic rejection (NRAD and fBOS) at UZLeuven.

These figures demonstrate that we are perfectly able to recruit the aforementioned numbers of patients for our study.

PART III. ENCLOSURES

1. Administrative data

Project coördinators:

Geert M Verleden, MD, PhD.

Division of Pulmonary Medicine

Head of the Lung Transplant Unit at UZ Gasthuisberg. Leuven. Belgium

Project leaders:

Bart M Vanaudenaerde (KULeuven; FWO postdoc)

Stijn Verleden (KULeuven; PhD student)

Address:

Katholieke Universiteit Leuven.

Department of Internal Medicine - Division of Pulmonary Medicine

Lung Transplantation Unit

University Hospital Gasthuisberg

Herestraat 49, B-3000 Leuven, Belgium

Phone number: +32 16 330195

Fax number: +32 16 347124

E-mail: Geert.Verleden@uzleuven.be

Bart.Vanaudenaerde@med.kuleuven.be

2. Declaration

On separate page

3. Official approval by the ethical commission:

Both arms of the study have been prepared separately and approved by the ethical comity of the University Hospital of Leuven, het Belgium Federal Agency for Medicines and Health Products (FAGG) and registrated internationally at eudraCT and clinicaltrial.gov. Important codes are for:

The vitamin D study:

EudraCT	2010-022027-30
Local ethical board	S52577 and ML6738
FAGG	AFMPS/R&D/CED/asa/111589
Clinicaltrial.gov	NCT01212406

The montelukast study:

EudraCT	2010-021983-14
Local ethical board	S52575 and ML6739
FAGG	AFMPS/R&D/CED/asa/111590
Clinicaltrial.gov	NCT01211509

4. Other financial support:

This clinical study with an important research component is based on the extended expertise in a specific field of the project. This project is supported by the other projects within the lab certainly at the start as the studies are planned to start at 09/2010. But the requested funding is essential to accomplish the study.

5. Track record:

(i) Relevant publications:

Vos R, Vanaudenaerde BM, Ottevaere A, Verleden SE, De Vleeschauwer SI, Willems-Widyastuti A, Wauters S, Van Raemdonck DE, Nawrot TS, Dupont LJ, Verleden GM. Long-term azithromycin therapy for bronchiolitis obliterans syndrome: Divide and conquer? J Heart Lung Transplant. 2010 Jul 7.(Epub ahead of print)

Vos R, Vanaudenaerde BM, Verleden SE, De Vleeschauwer SI, Willems-Widyastuti A, Van Raemdonck DE, Schoonis A, Nawrot TS, Dupont LJ, Verleden GM. A randomized placebo-

controlled trial of azithromycin to prevent bronchiolitis obliterans syndrome after lung transplantation. Eur Respir J. 2010 Jun 18. [Epub ahead of print]

Verleden GM, Vos R, De Vleeschauwer SI, Willems-Widyastuti A, Verleden SE, Dupont LJ, Van Raemdonck DE, Vanaudenaerde BM. Obliterative bronchiolitis following lung transplantation: from old to new concepts? Transpl Int. 2009: Aug;22(8):771-9.

Delaere P, Vranckx J, Verleden G, De Leyn P, Van Raemdonck D; Leuven Tracheal Transplant Group. Tracheal allotransplantation after withdrawal of immunosuppressive therapy. N Engl J Med. 2010; 362(2): 138-45.

Vanaudenaerde BM, Meyts I, Vos R, Geudens N, De Wever W, Verbeken EK, Van Raemdonck DE, Dupont LJ, Verleden GM. A dichotomy in bronchiolitis obliterans syndrome after lung transplantation revealed by azithromycin therapy. Eur Respir J. 2008 Oct;32(4):832-43.

Vanaudenaerde BM, De Vleeschauwer SI, Vos R, Meyts I, Bullens DM, Reynders V, Wuyts WA, Van Raemdonck DE, Dupont LJ, Verleden GM. The role of the IL23/IL17 axis in bronchiolitis obliterans syndrome after lung transplantation. Am J Transplant. 2008 Sep;8(9):1911-20.

Vos R, Vanaudenaerde BM, Geudens N, Dupont LJ, Van Raemdonck DE, Verleden GM. Pseudomonal airway colonisation: risk factor for bronchiolitis obliterans syndrome after lung transplantation? Eur Respir J. 2008 May;31(5):1037-45.

Blondeau K, Mertens V, Vanaudenaerde BA, Verleden GM, Van Raemdonck DE, Sifrim D, Dupont LJ. Gastro-oesophageal reflux and gastric aspiration in lung transplant patients with or without chronic rejection. Eur Respir J. 2008 ; Apr;31(4):707-13.

Vos R, Vanaudenaerde BM, Dupont LJ, Van Raemdonck DE, Verleden GM. Transient airway colonization is associated with airway inflammation after lung transplantation. Am J Transplant. 2007 May;7(5):1278-87.

Vanaudenaerde BM, Wuyts WA, Geudens N, Dupont LJ, Schoofs K, Smeets S, Van Raemdonck DE, Verleden GM. Macrolides inhibit IL17-induced IL8 and 8-isoprostane release from human airway smooth muscle cells. Am J Transplant. 2007; Jan;7(1):76-82.

Verleden GM, Vanaudenaerde BM, Dupont LJ, Van Raemdonck DE. Azithromycin reduces airway neutrophilia and interleukin-8 in patients with bronchiolitis obliterans syndrome. Am J Respir Crit Care Med. 2006 Sep 1;174(5):566-70.

Verleden GM, Dupont LJ. Inhaled cyclosporine in lung transplantation. N Engl J Med. 2006 Apr 20;354(16):1752-3

<u>(ii) awards:</u>

"ERS Young scientist sponsorship" of the ERS congress in Vienna 2003	(750 euro)
"ERS Young scientist sponsorship" of the ERS congress in Copenhagen 2005	(750 euro)
"Lung transplantation best abstract award" of the ERS congress, Stockholm 2007	(3000 euro)
"Best Thesis of 2007 award" of the BTS congress, Woluwe 2008	(1000 euro)
"GlaxoSmithKline award in Pneumologie" of the BVP meeting, Woluwe 2008	(1500 euro)
"Jacqueline Bernheim" Prize of the Society of Heart Surgery Belgium, 2008	(10000 euro)
"Lung transplantation best abstract award" of the ERS congress, Berlin 2008	(3000 euro)
"ERS Young scientist sponsorship" of the ERS congress in Berlin 2008	(750 euro)
"De Falloise Award" of the Belgian Lung and Tuberculosis Association 2008	(2000 euro)
"R. Pauwels Award" of the Belgian Society of Pneumology 2008	(2500 euro)
"Lung transplantation best abstract award" of the ERS congress, Vienna 2009	(3000 euro)
"Lung transplantation best abstract award" of the ERS congress Barcelona, 2010	(2000 euro)
"Henk Schippers Young Investigators Award" of Eurotransplant, 2010	(2500 euro)

REFERENCE LIST

(1) Sayegh MH, Carpenter CB. Transplantation 50 years later--progress, challenges, and promises. N Engl J Med 2004 Dec 23;351(26):2761-6.

(2) Estenne M, Kotloff RM. Update in transplantation 2005. Am J Respir Crit Care Med 2006 Mar 15;173(6):593-8.

(3) Estenne M, Maurer JR, Boehler A, Egan JJ, Frost A, Hertz M, et al. Bronchiolitis obliterans syndrome 2001: an update of the diagnostic criteria. J Heart Lung Transplant 2002 Mar;21(3):297-310.

(4) Vanaudenaerde BM, Meyts I, Vos R, Geudens N, De WW, Verbeken EK, et al. A dichotomy in bronchiolitis obliterans syndrome after lung transplantation revealed by azithromycin therapy. Eur Respir J 2008 Oct;32(4):832-43.

(5) Verleden GM, Vos R, De Vleeschauwer SI, Willems-Widyastuti A, Verleden SE, Dupont LJ, et al. Obliterative bronchiolitis following lung transplantation: from old to new concepts? Transpl Int 2009 Aug;22(8):771-9.

(6) Vanaudenaerde BM, Meyts I, Vos R, Geudens N, De WW, Verbeken EK, et al. A dichotomy in bronchiolitis obliterans syndrome after lung transplantation revealed by azithromycin therapy. Eur Respir J 2008 Oct;32(4):832-43.

(7) Vos R, Vanaudenaerde BM, Verleden SE, De Vleeschauwer SI, Willems-Widyastuti A, Van Raemdonck DE, et al. A randomized placebo-controlled trial of azithromycin to prevent bronchiolitis obliterans syndrome after lung transplantation. Eur Respir J 2010 Jun 18.

(8) Al MJ, Koopmanschap MA, van Enckevort PJ, Geertsma A, van der BW, de Boer WJ, et al. Cost-effectiveness of lung transplantation in The Netherlands: a scenario analysis. Chest 1998 Jan;113(1):124-30.

(9) van Enckevort PJ, Koopmanschap MA, TenVergert EM, Geertsma A, van der BW, de Boer WJ, et al. Lifetime costs of lung transplantation: estimation of incremental costs. Health Econ 1997 Sep;6(5):479-89.

(10) Sayegh MH, Carpenter CB. Transplantation 50 years later--progress, challenges, and promises. N Engl J Med 2004 Dec 23;351(26):2761-6.

(11) Boehler A, Estenne M. Post-transplant bronchiolitis obliterans. Eur Respir J 2003 Dec;22(6):1007-18.

(12) Christie JD, Edwards LB, Aurora P, Dobbels F, Kirk R, Rahmel AO, et al. The Registry of the International Society for Heart and Lung Transplantation: Twenty-sixth Official Adult Lung and Heart-Lung Transplantation Report-2009. J Heart Lung Transplant 2009 Oct;28(10):1031-49.

(13) Vos R, Vanaudenaerde BM, Verleden SE, De Vleeschauwer SI, Willems-Widyastuti A, Van Raemdonck DE, et al. A randomized placebo-controlled trial of azithromycin to prevent bronchiolitis obliterans syndrome after lung transplantation. Eur Respir J 2010 Jun 18.

(14) Verleden GM. Bronchiolitis obliterans syndrome after lung transplantation: medical treatment. Monaldi Arch Chest Dis 2000 Apr;55(2):140-5.

(15) Vanaudenaerde BM, Meyts I, Vos R, Geudens N, De WW, Verbeken EK, et al. A dichotomy in bronchiolitis obliterans syndrome after lung transplantation revealed by azithromycin therapy. Eur Respir J 2008 Oct;32(4):832-43.

(16) Vanaudenaerde BM, Meyts I, Vos R, Geudens N, De WW, Verbeken EK, et al. A dichotomy in bronchiolitis obliterans syndrome after lung transplantation revealed by azithromycin therapy. Eur Respir J 2008 Oct;32(4):832-43.

(17) Corris PA, Christie JD. Update in transplantation 2006. Am J Respir Crit Care Med 2007 Mar 1;175(5):432-5.

(18) Gottlieb J, Mattner F, Weissbrodt H, Dierich M, Fuehner T, Strueber M, et al. Impact of graft colonization with gram-negative bacteria after lung transplantation on the development of bronchiolitis obliterans syndrome in recipients with cystic fibrosis. Respir Med 2009 May;103(5):743-9.

(19) Botha P, Archer L, Anderson RL, Lordan J, Dark JH, Corris PA, et al. Pseudomonas aeruginosa colonization of the allograft after lung transplantation and the risk of bronchiolitis obliterans syndrome. Transplantation 2008 Mar 15;85(5):771-4.

(20) Vos R, Vanaudenaerde BM, Geudens N, Dupont LJ, Van Raemdonck DE, Verleden GM. Pseudomonal airway colonisation: risk factor for bronchiolitis obliterans syndrome after lung transplantation? Eur Respir J 2008 May;31(5):1037-45.

(21) Blondeau K, Mertens V, Vanaudenaerde BA, Verleden GM, Van Raemdonck DE, Sifrim D, et al. Gastro-oesophageal reflux and gastric aspiration in lung transplant patients with or without chronic rejection. Eur Respir J 2008 Apr;31(4):707-13.
(22) D'Ovidio F, Mura M, Tsang M, Waddell TK, Hutcheon MA, Singer LG, et al. Bile acid aspiration and the development of bronchiolitis obliterans after lung transplantation. J Thorac Cardiovasc Surg 2005 May;129(5):1144-52.
(23) Vos R, De VK, Schaevers V, Schoonis A, Lemaigre V, Dobbels F, et al. Smoking resumption after lung transplantation: a sobering truth. Eur Respir J 2010 Jun;35(6):1411-3.
(24) Dupont LJ, Dewandeleer Y, Vanaudenaerde BM, Van Raemdonck DE, Verleden GM. The pH of exhaled breath condensate of patients with allograft rejection after lung transplantation. Am J Transplant 2006 Jun;6(6):1486-92.
(25) Vos R, Cordemans C, Vanaudenaerde BM, De Vleeschauwer SI, Schoonis A, Van Raemdonck DE, et al. Exhaled carbon monoxide as a noninvasive marker of airway neutrophilia after lung transplantation. Transplantation 2009 May 27;87(10):1579-83.
(26) Vos R, Vanaudenaerde BM, De Vleeschauwer SI, Willems-Widyastuti A, Scheers H, Van Raemdonck DE, et al. Circulating and intrapulmonary C-reactive protein: a predictor of bronchiolitis obliterans syndrome and pulmonary allograft outcome. J Heart Lung Transplant 2009 Aug;28(8):799-807.
(27) Vos R, Vanaudenaerde BM, Verleden SE, De Vleeschauwer SI, Willems-Widyastuti A, Van Raemdonck DE, et al. Bronchoalveolar lavage neutrophilia in acute lung allograft rejection and lymphocytic bronchiolitis. J Heart Lung Transplant 2010 Jul 27.
(28) Dupont LJ, Dewandeleer Y, Vanaudenaerde BM, Van Raemdonck DE, Verleden GM. The pH of exhaled breath condensate of patients with allograft rejection after lung transplantation. Am J Transplant 2006 Jun;6(6):1486-92.
(29) Vos R, Cordemans C, Vanaudenaerde BM, De Vleeschauwer SI, Schoonis A, Van Raemdonck DE, et al. Exhaled carbon monoxide as a noninvasive marker of airway neutrophilia after lung transplantation. Transplantation 2009 May 27;87(10):1579-83.
(30) Vos R, Vanaudenaerde BM, De Vleeschauwer SI, Willems-Widyastuti A, Scheers H, Van Raemdonck DE, et al. Circulating and intrapulmonary C-reactive protein: a predictor of bronchiolitis obliterans syndrome and pulmonary allograft outcome. J Heart Lung Transplant 2009 Aug;28(8):799-807.
(31) Klepetko W, Estenne M, Glanville A, Verleden G, Aubert JD, Sarahrudi K, et al. A multicenter study to assess outcome following a switch in the primary immunosuppressant from cyclosporin (CYA) to tacrolimus (TAC) in lung recipients. J Heart Lung Transplant 2001 Feb;20(2):208.
(32) Sarahrudi K, Estenne M, Corris P, Niedermayer J, Knoop C, Glanville A, et al. International experience with conversion from cyclosporine to tacrolimus for acute and chronic lung allograft rejection. J Thorac Cardiovasc Surg 2004 Apr;127(4):1126-32.
(33) Vos R, Vanaudenaerde BM, Verleden SE, De Vleeschauwer SI, Willems-Widyastuti A, Van Raemdonck DE, et al. A randomized placebo-controlled trial of azithromycin to prevent bronchiolitis obliterans syndrome after lung transplantation. Eur Respir J 2010 Jun 18.
(34) Snell GI, Valentine VG, Vitulo P, Glanville AR, McGiffin DC, Loyd JE, et al. Everolimus versus azathioprine in maintenance lung transplant recipients: an international, randomized, double-blind clinical trial. Am J Transplant 2006 Jan;6(1):169-77.

(35) Janssens W, Bouillon R, Claes B, Carremans C, Lehouck A, Buysschaert I, et al. Vitamin D deficiency is highly prevalent in COPD and correlates with variants in the vitamin D-binding gene. Thorax 2010 Mar;65(3):215-20.
(36) Janssens W, Lehouck A, Carremans C, Bouillon R, Mathieu C, Decramer M. Vitamin D beyond bones in chronic obstructive pulmonary disease: time to act. Am J Respir Crit Care Med 2009 Apr 15;179(8):630-6.
(37) Moro JR, Iwata M, von Andriano UH. Vitamin effects on the immune system: vitamins A and D take centre stage. Nat Rev Immunol 2008 Sep;8(9):685-98.
(38) Yoshisue H, Kirkham-Brown J, Healy E, Holgate ST, Sampson AP, Davies DE. Cysteinyl leukotrienes synergize with growth factors to induce proliferation of human bronchial fibroblasts. J Allergy Clin Immunol 2007 Jan;119(1):132-40.
(39) Ramsay CF, Sullivan P, Gizycki M, Wang D, Swern AS, Barnes NC, et al. Montelukast and bronchial inflammation in asthma: a randomised, double-blind placebo-controlled trial. Respir Med 2009 Jul;103(7):995-1003.
(40) Ramsay CF, Sullivan P, Gizycki M, Wang D, Swern AS, Barnes NC, et al. Montelukast and bronchial inflammation in asthma: a randomised, double-blind placebo-controlled trial. Respir Med 2009 Jul;103(7):995-1003.
(41) Izumo T, Kondo M, Nagai A. Cysteinyl-leukotriene 1 receptor antagonist attenuates bleomycin-induced pulmonary fibrosis in mice. Life Sci 2007 Apr 24;80(20):1882-6.
(42) El-Swefy S, Hassanen SI. Improvement of hepatic fibrosis by leukotriene inhibition in cholestatic rats. Ann Hepatol 2009 Jan;8(1):41-9.
(43) Or R, Gesundheit B, Resnick I, Bitan M, Avraham A, Avgil M, et al. Sparing effect by montelukast treatment for chronic graft versus host disease: a pilot study. Transplantation 2007 Mar 15;83(5):577-81.
(44) Verleden GM. Bronchiolitis obliterans syndrome after lung transplantation: medical treatment. Monaldi Arch Chest Dis 2000 Apr;55(2):140-5.
(45) Glenville J. Pharmacokinetics of vitamin D toxicity. Am J Clin Nutr 2008;88:582S-6S.
